# Non-canonical BIM-regulated energy metabolism determines drug-induced liver necrosis

**DOI:** 10.1038/s41418-023-01245-7

**Published:** 2023-11-24

**Authors:** Rebekka Lambrecht, Franziska Rudolf, Anna-Katharina Ückert, Valentina C. Sladky, Truong San Phan, Jasmin Jansen, Samara Naim, Thomas Kaufmann, Adrian Keogh, Susanne Kirschnek, Aswin Mangerich, Florian Stengel, Marcel Leist, Andreas Villunger, Thomas Brunner

**Affiliations:** 1 Biochemical Pharmacology, Department of Biology, University of Konstanz, Universitätsstrasse 10, 78464 Konstanz, Germany; 2 In vitro Toxicology and Biomedicine, Department of Biology, University of Konstanz, Universitätsstrasse 10, 78464 Konstanz, Germany; 3 Institute for Developmental Immunology, Biocenter, Medical University of Innsbruck, Innrain 80, 6020 Innsbruck, Austria; 4 Biochemistry and Mass Spectrometry, Department of Biology, University of Konstanz, Universitätsstrasse 10, 78464 Konstanz, Germany; 5 Institute of Pharmacology, University of Bern, Inselspital, Bern University Hospital, INO-F, Freiburgstrasse 16C, 3010 Bern, Switzerland; 6 Visceral and Transplantation Surgery, Department of Clinical Research, Inselspital, Bern University Hospital, 3008 Bern, Switzerland; 7 Faculty of Medicine, Institute of Medical Microbiology and Hygiene, Medical Center, University of Freiburg, 79104 Freiburg, Germany; 8 Nutritional Toxicology, Institute of Nutritional Science, University of Potsdam, Arthur-Scheunert-Allee 114-116, 14558 Nuthetal, Germany; 9 The Research Center for Molecular Medicine (CeMM) of the Austrian Academy of Sciences, Lazarettgasse 14, 1090 Vienna, Austria; 10 Ludwig Boltzman Institute for Rare and Undiagnosed Diseases (LBI-RUD), Lazarettgasse 14, 1090 Vienna, Austria

**Keywords:** Cell biology, Gastrointestinal diseases

## Abstract

Paracetamol (acetaminophen, APAP) overdose severely damages mitochondria and triggers several apoptotic processes in hepatocytes, but the final outcome is fulminant necrotic cell death, resulting in acute liver failure and mortality. Here, we studied this switch of cell death modes and demonstrate a non-canonical role of the apoptosis-regulating BCL-2 homolog BIM/Bcl2l11 in promoting necrosis by regulating cellular bioenergetics. BIM deficiency enhanced total ATP production and shifted the bioenergetic profile towards glycolysis, resulting in persistent protection from APAP-induced liver injury. Modulation of glucose levels and deletion of Mitofusins confirmed that severe APAP toxicity occurs only in cells dependent on oxidative phosphorylation. Glycolytic hepatocytes maintained elevated ATP levels and reduced ROS, which enabled lysosomal recycling of damaged mitochondria by mitophagy. The present study highlights how metabolism and bioenergetics affect drug-induced liver toxicity, and identifies BIM as important regulator of glycolysis, mitochondrial respiration, and oxidative stress signaling.

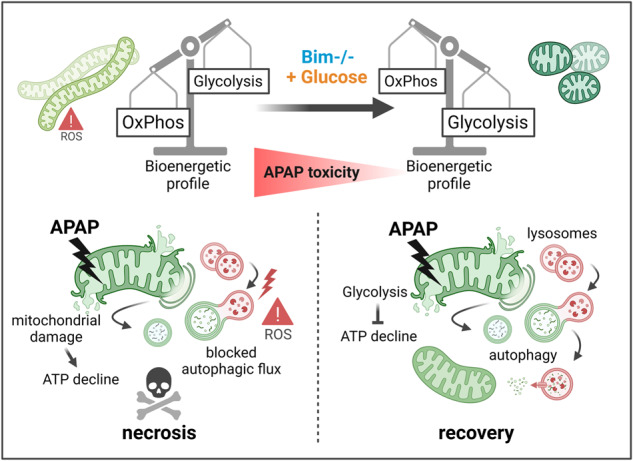

## Introduction

Cell death is broadly subdivided into apoptosis, a caspase-dependent form of programmed cell death, necrosis as an unintended and often deleterious cell death process, and additional forms of programmed cell death with necrotic features. While in a simplified view different forms of cell death proceed independently and mutually exclusive, there is accumulating evidence for extensive crosstalk between different cell death modalities [1], including non-canonical functions of cell death-regulating proteins in other forms of programmed cell death [2–4]. To understand how different forms of cell death crosstalk and how potential decision points lead to a switch between different types, we studied the prominent clinical phenomenon of Paracetamol-induced hepatocyte death and accompanied liver damage.

Paracetamol (acetaminophen, n-acetyl-p-aminophenol, APAP) intoxication severely damages the liver and represents the most frequent cause of acute liver failure (ALF) in developed countries [5]. APAP overdose saturates classical detoxification pathways and causes the accumulation of n-acetyl-p-benzoquinone (NAPQI), an intermediate from cytochrome P450 enzyme-dependent APAP metabolism [6]. NAPQI depletes glutathione (GSH) and forms covalent protein adducts, thereby interfering with numerous cellular processes, depleting ATP levels and provoking exceptionally high levels of reactive oxidative species (ROS) [7–9]. A vicious cycle of mitochondrial damage and oxidative stress represents the most fatal event promoting hepatocyte death [10]. Due to persistent controversies about the relevant intracellular processes in APAP-intoxicated hepatocytes, the GSH-precursor N-acetylcysteine is still the only approved antidote for AILI patients [11].

We chose APAP-induced liver injury (AILI) as model to study cell death decisions as the type of hepatocyte death upon APAP overdose is intriguing and highly controversial [12, 13]. Although APAP-intoxicated hepatocytes phenotypically die by oncotic necrosis, APAP initially triggers multiple stress programs, including processes attributed to apoptosis. Apoptotic mitochondrial outer membrane permeabilization (MOMP) contributes to APAP-induced loss of mitochondrial integrity, potentiating ATP depletion and ROS formation [14, 15]. The apoptosis-regulating B-cell lymphoma 2 (BCL-2) protein family facilitates MOMP and associated caspase activation [16], and was paradoxically shown to regulate APAP-induced necrosis. Briefly, deletion of each, pro-apoptotic BCL-2-associated X protein (BAX) [17], BCL-2-like protein 11 (BIM) [18], p53 upregulated modulator of apoptosis (PUMA) [19] and BH3 interacting-domain death agonist (BID) [20] protected from APAP-induced liver necrosis. In the present study, we investigated how these pro-apoptotic proteins regulate necrotic cell death during AILI with the aim to clarify the paradox of initial apoptotic processes that result in a necrotic outcome.

Our data provide strong evidence for a novel, non-canonical (apoptosis-independent) function of BIM in regulating mitochondrial dynamics and cellular energy metabolism, and thereby oncotic necrosis. BIM deletion shifted the energy production from mitochondria-dependent oxidative phosphorylation to glycolysis, which reduced the dependency on malfunctional mitochondria and allowed recovery after APAP intoxication. Remarkably, glucose administration likewise protected mice and cells from APAP-induced damage and may represent a novel treatment option during AILI.

## Results

### APAP induces apoptotic BH3-only proteins but fails to activate caspases

**Fig. 1:**
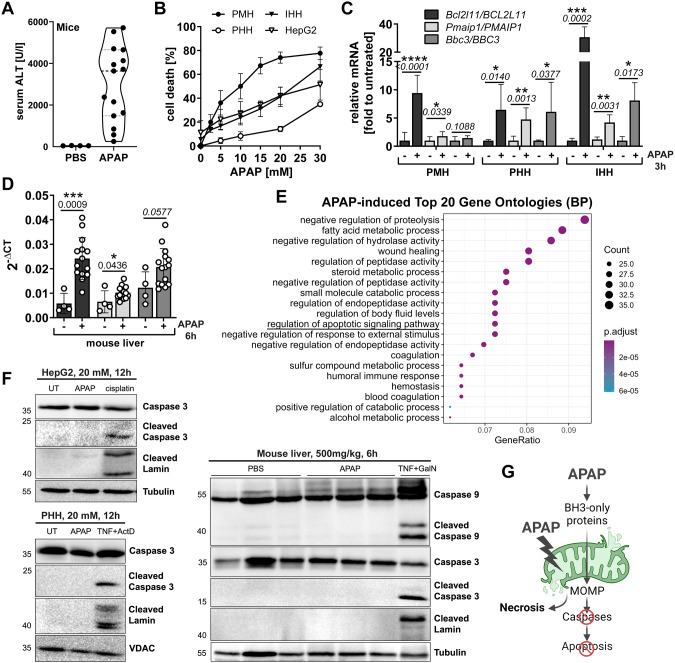
APAP induces apoptotic BH3-only proteins but fails to activate caspases. **A** Serum ALT of mice treated for 6 h with PBS or 500 mg/kg APAP i.p. Bold dotted line indicates median and weak dotted lines show quartiles. **B** MTT assay of primary murine (PMH) and human hepatocytes (PHH) treated for 16 h or 24 h, respectively, or FACS analysis of AnnexinV-FITC + IHH and HepG2 cells treated for 24 h. *n* = 5 (PMH), *n* = 2 (PHH), *n* = 13 (IHH), *n* = 4 (HepG2). **C**, **D** Transcript levels of mouse liver of mice treated as described in **A**, and of PMH, PHH and IHH treated for 3 h with 10 mM APAP (PMH) or with 20 mM APAP (PHH, IHH). *n* = 7 (PMH), *n* = 6 (PHH), *n* = 5 (IHH). **E** Top 20 gene ontologies of hepatocyte subset from mice treated as described in **A** generated from single cell RNA sequencing. BP biological function. **F** Western blot of ML of mice treated as described in **A** and of PHH and HepG2 treated with 20 mM APAP for 12 h. **G** Schematic illustration of APAP-induced apoptotic processes. Data points and/or bar graphs are mean +/− SD with *n* as independent biological replicates. Statistical significance was tested using unpaired Student’s *t* test (**C**, **D**).

To investigate APAP-induced liver damage and cell death, we made use of different experimental systems. Animals injected with APAP showed extensive liver damage, and ex vivo cultured murine and human hepatocytes, and the human hepatocyte cell lines IHH and HepG2 dose-dependently died in response to APAP treatment (Fig. 1A, B). This was accompanied by induction of the pro-apoptotic proteins BIM (*Bcl2l11*), NOXA (*Pmaip1*) and PUMA (*Bbc3*) in all models investigated (Fig. 1C, D, Supplementary Fig. S1A). Human cell lines responded generally slower to APAP than murine cells, as illustrated by delayed activation of the stress kinase c-Jun N-terminal kinase (JNK) (Supplementary Fig. S1B). Single-cell RNA sequencing data obtained from Kolodziejczyk et al. [21] of APAP-treated mice confirmed the dominant upregulation of apoptosis-related genes in the hepatocyte subset (Fig. 1E, Supplementary Fig. S1C, D). In addition, gene ontology (GO) analysis underpinned an important role of mitochondria and oxidative stress upon APAP (Supplementary Fig. S1D). However, despite the dominant apoptotic response after APAP treatment, hepatocytes failed to show apoptosis-related caspase activation and underwent cell death with a necrotic phenotype that was not prevented by caspase inhibition (Fig. 1F, G, Supplementary Fig. S1E–H).

### Depletion of BIM protects from APAP-induced liver necrosis

**Fig. 2:**
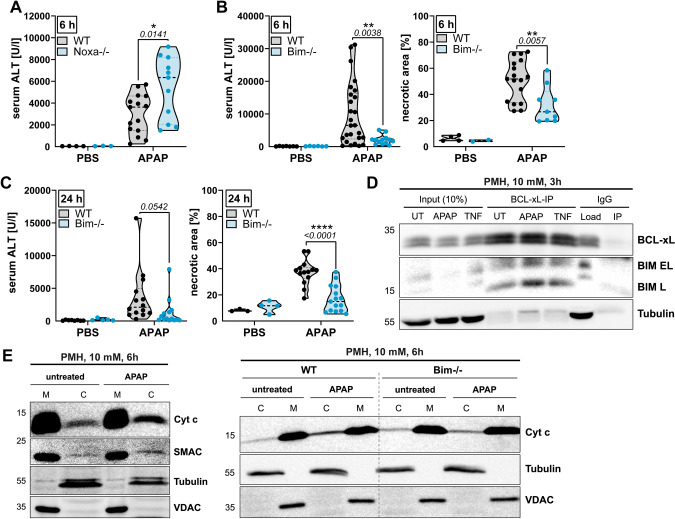
Depletion of BIM protects from APAP-induced liver necrosis. **A–C** Serum ALT and necrotic area scored from histology images (see Supplementary Fig. S3A) of WT, *Noxa*−/− and *Bim*−/− mice treated for 6 h with 500 mg/kg (**A**, **B**) or for 24 h with 300 mg/kg APAP (**C**). Bold dotted line indicates median and weak dotted lines show quartiles. Statistical significance was tested using Two-way ANOVA with Sidak’s multiple comparison test. **D** Western Blot of BCL-xL immunoprecipitation of primary murine hepatocytes (PMH) untreated (UT) or treated for 3 h with 10 mM APAP or 2 ng/ml murine TNF and 30 nM Actinomycin D (ActD). **E** Western Blot of cytosolic (C) and mitochondrial (M) fractions of PMH treated with 10 mM APAP for 6 h.

The BH3-only proteins BIM and PUMA had been previously implicated in AILI [19, 22]. Since we observed that NOXA was also strongly induced, we investigated the role and regulation of NOXA in AILI. PUMA and NOXA are major p53 targets in response to DNA damage [23]. APAP treatment led to DNA damage and promoted the upregulation of p53 target genes (Supplementary Fig. S2A, B), however, deleting p53 failed to prevent NOXA induction after APAP treatment (Supplementary Fig. S2C). Mice lacking p53 showed reduced, but not abolished NOXA transcription in response to APAP treatment (Supplementary Fig. S2D), indicating additional p53-independent processes regulating NOXA expression. APAP also led to activation of the stress kinases p38 and JNK (Supplementary Fig. S2E). While deficiency in p38 did not impact NOXA induction (Supplementary Fig. S2F), pharmacological inhibition of JNK strongly reduced APAP-induced NOXA expression in IHH and HepG2 cells (Supplementary Fig. S2G). Interestingly, NOXA deletion in mice did rather result in increased liver damage, whereas BIM deficiency clearly protected mice from AILI (Fig. 2A-C, Supplementary Fig. S3A). Of note, NOXA-deficient livers did not display higher transcripts of BIM or PUMA (Supplementary Fig. S3B), arguing against compensatory effects. Although NOXA and BIM exhibited their canonical function by binding MCL-1 and BCL-x_L_ (Fig. 2D, Supplementary Fig. S3C, D) and promoting MOMP-related cytochrome c release (Fig. 2E), hepatocytes failed to activate caspases in response to APAP, in vitro and in vivo (Fig. 1). Thus, APAP only initiates signaling processes potentially leading to apoptosis, but cell death is not executed by apoptosis. The presented data suggest a unique, non-canonical role of BIM in regulation APAP-initiated necrosis as the protective effect of BIM deletion is not reproduced by NOXA deletion, and furthermore is long-lasting compared to the only temporary protection seen by deleting BAX [17]. The fact that BIM deficiency, but not BAX deficiency, protects enduringly in the same experimental setup suggests a MOMP-independent function of BIM. This proposed non-apoptotic function of BIM likely runs in parallel with its canonical function promoting MOMP during APAP intoxication.

### BIM contributes to APAP-induced mitochondrial damage and energy crisis

**Fig. 3:**
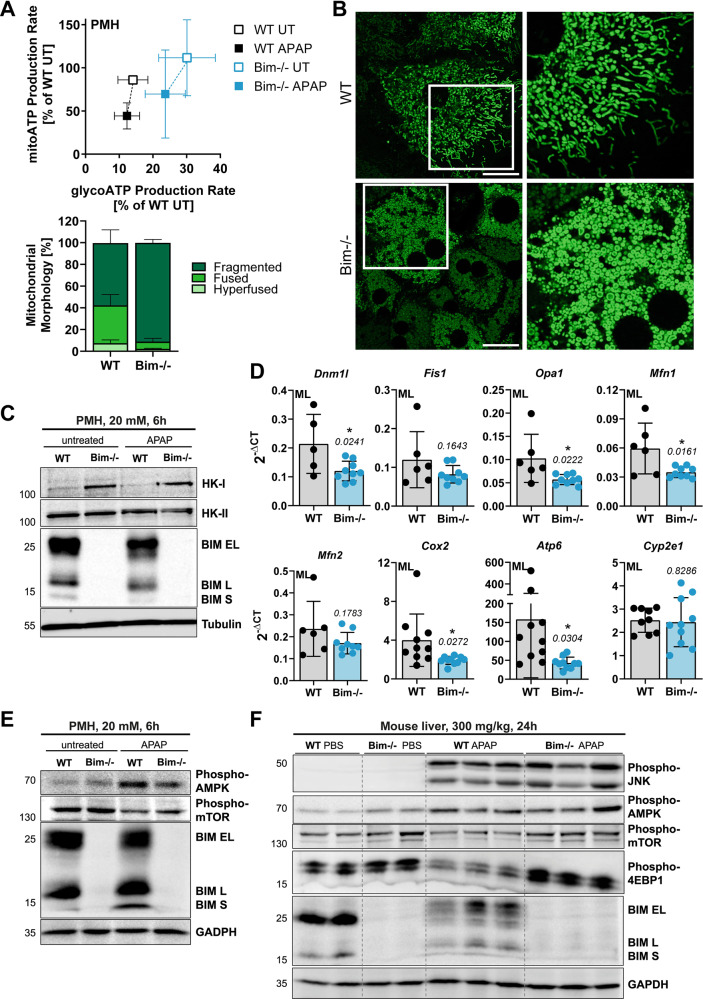
BIM contributes to APAP-induced mitochondrial damage and energy crisis. **A** Energy Map of Seahorse Induced ATP Rate Assay with medium (UT) or APAP injection (final 15 mM APAP) of primary murine hepatocytes (PMH), *n* = 4–8. Data normalized to sum of mito- and gylcoATP of WT untreated PMH. **B** Representative confocal images of MitoTracker Green-stained PMH and quantification of mitochondria morphology based on length to width ratio, *n* = 3. Scale bar 20 µm. **C** Western Blot of PMH treated with 20 mM APAP for 6 h. **D** Transcript levels of untreated murine livers (ML) of WT and *Bim*−/− mice. **E, F** Western Blot of PMH treated with 20 mM APAP for 6 h (**E**) and ML of mice treated with 300 mg/kg APAP for 24 h (**F**). Data points and/or bar graphs are mean +/− SD with *n* as independent biological replicates. Statistical significance was tested using unpaired Student’s *t* test (**D**).

Dysfunctional mitochondria during APAP toxicity are fatal as they provoke a cellular energy crisis forcing the cell into necrosis [8, 15]. BIM-deficient hepatocytes showed attenuated cytochrome c release (Fig. 2E) and reduced loss of mitochondrial membrane potential after APAP challenge (Supplementary Fig. S4A), indicating lower sensitivity towards mitochondrial effects of APAP. Both can be attributed to BIM’s canonical, i.e. MOMP-promoting, function. However, we further hypothesized that BIM possesses an additional, non-apoptotic mode of action related to mitochondrial energy metabolism. Remarkably, we found that BIM-deficient hepatocytes produce more ATP, even under steady state conditions, and were thus better positioned facing the APAP-induced drastic ATP depletion (Fig. 3A, Supplementary Fig. S4B). In detail, untreated BIM-deficient hepatocytes displayed a greater increase in glycolytic than mitochondrial energy production (from 14% to 30% glycoATP, from 86% to 112% mitoATP, compared to wild type hepatocytes), which might represent the critical advantage as APAP mainly targets mitochondrial ATP production (Fig. 3A, Supplementary Fig. S4B). This was underpinned by more lactate production in BIM-deficient hepatocytes (Supplementary Fig. S4C). Remarkably, this bioenergetic profile shift was accompanied by a pronounced change from fused, elongated mitochondria in wild type cells to increased mitochondrial fission in BIM-deficient hepatocytes (Fig. 3B), representing a hallmark of glycolytically active cells [24, 25]. Increased mitochondrial fission and increased glycolysis upon BIM depletion was confirmed in HepG2 and IHH cells (Supplementary Fig. S3D–G). In contrast to BAX and BAK-deficient cells, HepG2 BIM−/− cells showed still cytochrome c release, which further strengthens our hypothesis of a MOMP-unrelated protection in BIM−/− cells (Supplementary Fig. S3E). Intriguingly, additional BAX and BAK-deficiency did not reverse the phenotype of BIM−/− cells likely because BAX/BAK depletion caused itself an increase in glycolysis and fragmentation of mitochondria (Supplementary Fig. S3F, G). BAX and BAK were shown to regulate mitochondrial morphology independent of apoptosis induction by interacting with Mitofusin 2 and DRP1 [26–28]. We did not find evidence that BIM interacts with DRP1 or Mitofusin 2 (Supplementary Fig. S4H). Furthermore, increased glycolysis in BIM-deficient cells correlated with elevated Hexokinase-I (HK-I) proteins levels in cell lines, hepatocytes and mouse livers, which importantly was not present in BAX/BAK-deficient cells (Fig. 3C, Supplementary Fig. S4I). Taken together, this reduces the likelihood that mitochondrial fragmentation in BIM-deficient cells is based on the same mechanism as in BAX/BAK-deficient cells, and suggests that the fragmentation is a consequence of increased glycolysis. In all experimental systems, APAP caused fragmentation of mitochondria (not shown), which is consistent with earlier results showing that mitochondrial damage is accompanied by mitochondrial fission and swelling [25, 28]. Interestingly, BIM-deficient livers showed in general reduced mitochondrial transcripts, while the cytosolic *Cyp2e1* expression (metabolizing APAP to NAPQI) was unaffected (Fig. 3D). In addition, Parkin protein levels were remarkably increased in BIM-deficient cells across different organs (Supplementary Fig. S4G, H). The fragmented mitochondrial morphology together with the elevated mitophagy and glycolysis markers suggest a reduced dependency on mitochondria in cells lacking BIM. BIM-deficient HepG2 cells and splenocytes isolated from *Bim*−/− mice showed reduced basal mitochondrial membrane potential compared to wild type cells (Supplementary Fig. S4J), supporting the hypothesis of a reduced dependency on mitochondrial energy production. This correlated with lower sensitivity of BIM-deficient splenocytes to the mitochondrial toxin Rotenone (Supplementary Fig. S4K, L), similar to the protection seen in APAP-treated hepatocytes (Fig. 3A, Supplementary Fig. S4B). The beneficial energy status during APAP intoxication via increased mitochondria-independent energy production was confirmed by attenuated AMPK and stabilized mTOR phosphorylation in APAP-treated BIM-deficient hepatocytes and mice (Fig. 3E, F, Supplementary Fig. S4M).

### Sensitivity to APAP-induced toxicity is dependent on mitochondrial energy production

**Fig. 4:**
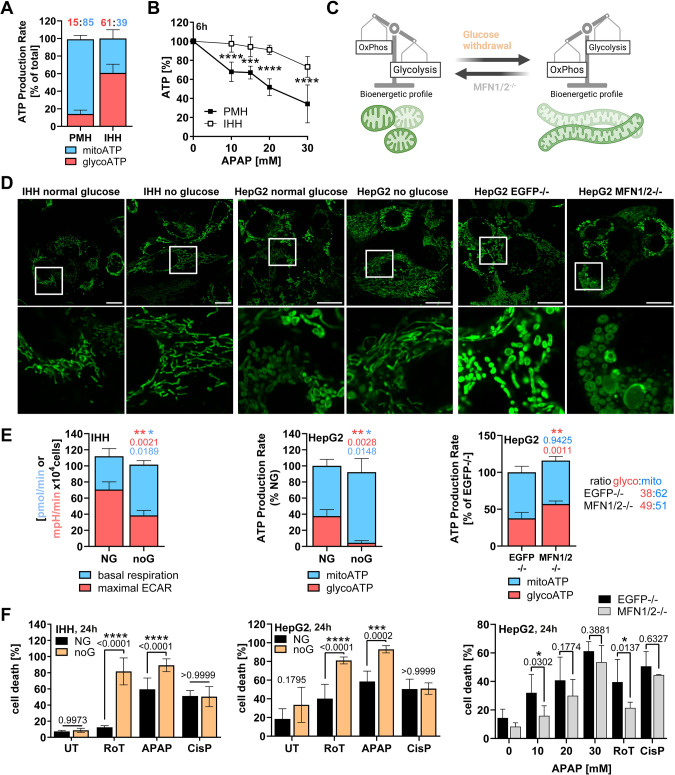
Sensitivity to APAP-induced toxicity depends on mitochondrial energy production. **A** Seahorse Basal ATP Rate Assay of untreated primary murine hepatocytes (PMH) and IHH with glyco/mitoATP ratio above, *n* = 6–8. **B** CellTiter-Glo Assay of PMH and IHH cells treated for 6 h, *n* = 4–9. **C** Working hypothesis: Glucose withdrawal shifts cell lines to oxidative phosphorylation (OxPhos) dependency and elongated mitochondria (green), while MFN1/2 knockout reverses the phenotype. **D** Representative confocal images of MitoTracker Green-stained untreated IHH and HepG2 cultured in different glucose concentrations. Scale bar 20 µm. **E** Seahorse Mito Stress Test Assay of IHH (left) or Basal ATP Rate Assay of HepG2 (mid+right) cultured in normal (NG) or no glucose (noG). Basal respiration and maximal ECAR surrogate for mitochondrial and glycolytic activity. Statistical significance tested by comparing mitoATP and glycoATP independently. *n* = 3 (all). **F** FACS analysis of AnnexinV-FITC + IHH and HepG2 treated with 20 mM APAP for 24 h and cultured in NG or noG. *n* = 3–6 (left), *n* = 4 (mid), *n* = 4 (right). Data points and/or bar graphs are mean +/− SD with *n* as independent biological replicates. Statistical significance was tested using unpaired Student’s *t* test (**E**) or Two-way ANOVA with Sidak’s multiple comparison test (**B**, **F**).

To test our hypothesis that BIM-deficient hepatocytes are protected from APAP due to their glycolytic energy phenotype, we analyzed IHH cells, which possess a lower dependency on mitochondrial energy production compared to primary hepatocytes (Fig. 4A). This difference had functional consequences as seen by the opposing sensitivity to glycolysis or mitochondrial complex inhibitors (Supplementary Fig. S5A), and by IHH cells being less sensitive to APAP-induced ATP loss (Fig. 4B). To investigate whether the cellular energy phenotype defines APAP sensitivity, we forced IHH and HepG2 cells to different bioenergetic profiles by culturing them in media without glucose or by deleting Mitofusin-1 and -2 (MFN1/2) proteins (Fig. 4C) [24, 29]. Starved cells showed characteristic elongation of mitochondria (Fig. 4D, left+mid) and increased mitochondria-dependent energy production (Fig. 4E, left+mid, Supplementary Fig. S5B, C). In contrast, MFN1/2 deficiency resulted in mitochondrial hyperfragmentation and elevated glycolysis (Fig. 4D, E, right). Strikingly, increased oxidative phosphorylation in starved cells drastically increased their sensitivity towards the mitochondria-damaging substances Rotenone and APAP, while leaving cisplatin-induced cell death unaffected (Fig. 4F, Supplementary Fig. S5D). Vice versa, increased glycolysis in *MFN1/2*−/− cells reduced sensitivity to APAP and Rotenone (Fig. 4F). These data point out that APAP toxicity critically relies on the cell’s bioenergetic profile, precisely on the dependency on mitochondria as the primary energy supplier.

### Glycolysis-mediated energy production rescues from APAP toxicity

**Fig. 5:**
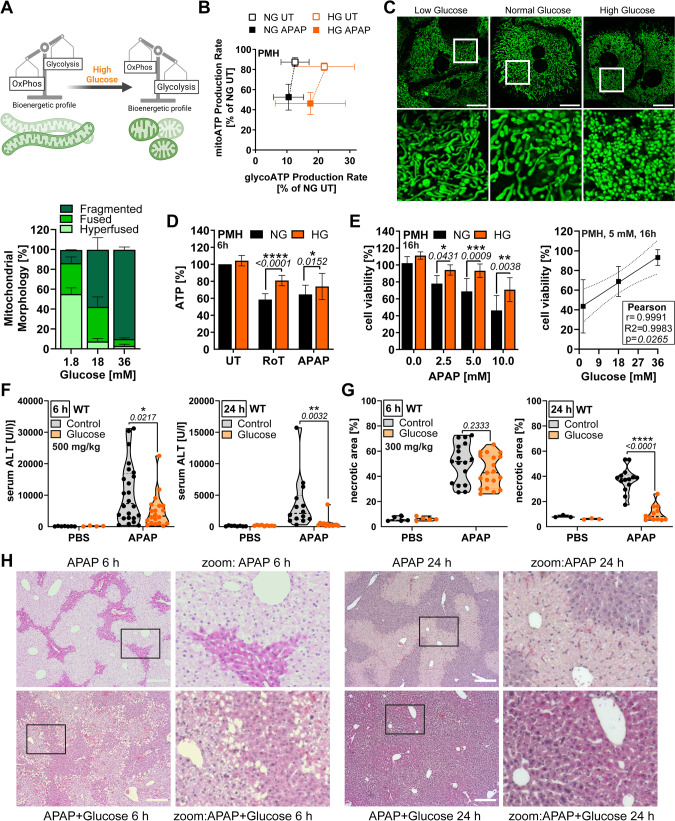
Glycolysis-mediated energy production rescues from APAP toxicity. **A** Working hypothesis: Glucose administration shifts primary cells to glycolysis dependency and fragmented mitochondria (green). **B** Energy Map of Seahorse Induced ATP Rate Assay with medium (untreated, UT) or APAP injection (final 15 mM) of primary murine hepatocytes (PMH) cultured in normal glucose (NG) or high glucose (HG), *n* = 3–8. Data normalized to sum of mito- and gylcoATP of NG-conditioned, untreated PMH. **C** Representative confocal images of MitoTracker Green-stained PMH cultured in different glucose concentrations with quantification of mitochondria morphology based on length to width ratio, *n* = 3. Scale bar 20 µm. **D** CellTiter-Glo Assay of PMH cultured in NG or HG and treated with 10 mM APAP or Rotenone for 6 h, *n* = 3. **E** MTT assay of PMH cultured in NG or HG and treated with APAP for 16 h, *n* = 7. **F**–**H** Serum ALT and necrotic area of scored from H&E-stained histology images of mice treated for 6 h with 500 mg/kg (left) or for 24 h with 300 mg/kg APAP (right) with optional injection of 2.5 g/kg glucose 1 h prior to APAP. Bold dotted line indicates median and weak dotted lines show quartiles. Scale bars 250 µm (**G**). Data points and/or bar graphs are mean +/− SD with *n* as independent biological replicates. Statistical significance was tested using Two-way ANOVA with Sidak’s multiple comparison test (**D**–**G**).

To answer whether shifting primary cells from mitochondria-dependent to -independent energy production causes a protective effect, we cultured murine hepatocytes in high glucose (HG) media, which elevated glycolysis, without affecting oxidative phosphorylation (Fig. 5A, B, Supplementary Fig. S6A, B). Nutrient excess resulted in mitochondrial fragmentation, while starved hepatocytes displayed hyperfused mitochondria (Fig. 5C). Of note, the ratio of fused/fragmented mitochondria in the high glucose condition very much resembled that of BIM-deficient cells (Fig. 3B). Similar to BIM- or MFN1/2-deficient cells, high glucose conditioning decreased the sensitivity towards mitochondrial-damaging agents Rotenone and APAP (Fig. 5D, E, Supplementary Fig. S6C–E), while culturing in low glucose increased the sensitivity to APAP-induced necrosis (Supplementary Fig. S6F), generating a strong correlation between glucose concentration and APAP sensitivity (Fig. 5E). Cotreatment with the glycolysis inhibitor 2-Deoxyglucose abolished the glucose-mediated protection, reinforcing the link between APAP sensitivity and glycolytic ATP production (Supplementary Fig. S6D). The protective effect of glucose could be also confirmed in primary human hepatocytes (Supplementary Fig. S6E). Importantly, in vivo glucose administration 1 h prior to treatment with APAP for 6 h or 24 h resulted in increased blood sugar levels (Supplementary Fig. S6G) and a significant reduction in serum ALT and necrotic areas (Fig. 5F–H). Interestingly, glucose seemed to be more efficient in preventing toxicity at lower APAP doses and longer treatment time. Importantly, caspase activation could not be restored under any of these conditions, and also not in livers of BIM-deficient mice (Supplementary Fig. S7). This challenges the current dogma that APAP-induced cell death is necrotic because of the emerging energy crisis, but rather suggests additional APAP-induced processes that prevent caspase activation.

### Fasting is beneficial by priming for antioxidant and autophagy responses prior to APAP intoxication

**Fig. 6:**
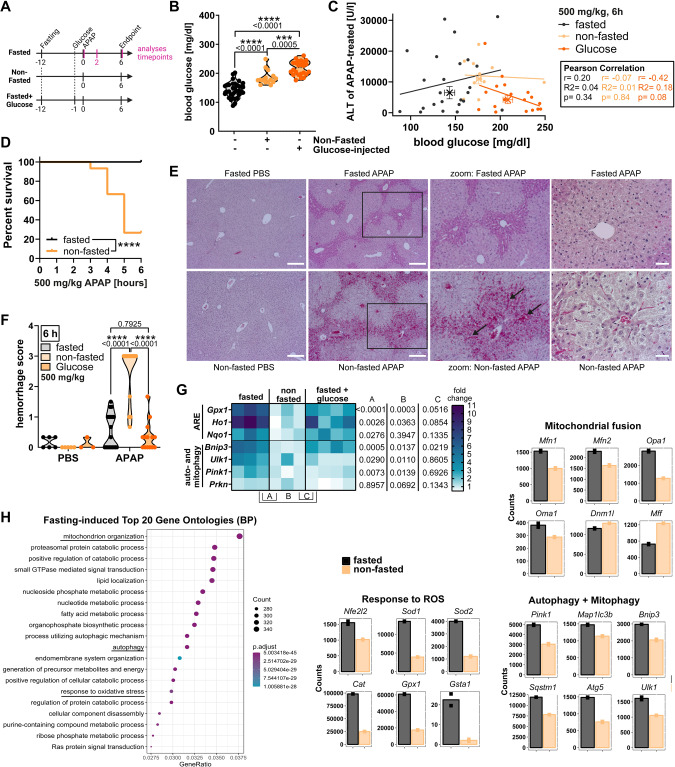
Fasting is beneficial by priming for antioxidant and autophagy responses prior to APAP intoxication. **A** Illustrated timeline of animal experiments with fasting 12 h prior to PBS/APAP injection and glucose injection 1 h prior to PBS/APAP injection. Glucose levels in (B + C) were measured at t = 0. Gene expression in (G) was measured at t = 2. ALT and liver damage was measured at t = 6. **B** Blood glucose levels of mice 1 h after 2.5 g/kg glucose injection, and of mice non-fasted or fasted overnight. Bold dotted line indicates median and weak dotted lines quartiles. **C** Correlation between serum ALT of mice treated with 500 mg/kg APAP for 6 h and respective blood glucose prior to APAP treatment. Crosses indicate group median +/− SEM. Statistical significance and linear fit was calculated by Pearson correlation. **D** Kaplan–Meier survival curve of fasted and nonfasted mice treated with APAP (6 h, 500 mg/kg APAP), *n* = 26 (fasted), *n* = 15 (non-fasted). Statistical significance was calculated using Gehan–Breslow–Wilcoxon test. **E**, **F** Representative images of H&E-stained liver sections and hemorrhage scoring of fasted or non-fasted mice treated with 500 mg/kg APAP for 6 h. Scale bars 250 µm (left), 50 µm (right). **G** Transcript levels of livers from nonfasted/fasted mice 2 h after glucose injection. Heatmap shows fold change to nonfasted and respective statistical significances on the right. **H** Fasting-induced gene ontologies of hepatocyte DEGs (left) and gene expression counts of selected DEGs (right) from overnight fasted mice analyzed by hepatocyte bulkRNA sequencing. BP biological process. Data points and/or bar graphs are mean +/− SD with *n* as independent biological replicates. Statistical significance was tested using Two-way ANOVA with Sidak’s multiple comparison test (**B**, **F**, **G**).

To assess whether the nutritional status, and specifically the blood glucose levels, determine the degree of AILI, as we would predict from our in vitro experiments (Fig. 5D), we compared APAP sensitivity of fasted and non-fasted mice (Fig. 6A). Prior to APAP challenge, non-fasted mice displayed intermediate blood glucose and energy status as visualized by the degree of AMPK phosphorylation (Fig. 6B, Supplementary Fig. S8A, B). Fasting of animals before APAP injection is a standard procedure and seen as pre-requisite for AILI induction ever since AILI animal models were studied [30, 31]. Thus, rather surprising, we saw no correlation between blood glucose levels and liver damage (Fig. 6C), and although serum ALT levels and necrotic area were not significantly different (Supplementary Fig. S8C, D), non-fasted mice died earlier than their fasted littermates (Fig. 6D). The early mortality of non-fasted mice seemed to result from more progressed hepatocyte necrosis accompanied by extensive hemorrhage in the surrounding tissue (Fig. 6E, F). To understand the reason behind this observation, we compared gene expression of fasted, non-fasted and fasted animals that received glucose injection, in the absence of APAP treatment. Fasting resulted in the upregulation of antioxidant response (ARE) and autophagy genes, and additional glucose treatment reduced these effects (Fig. 6G). Notably, the fasting-mediated upregulation of autophagy genes was persistent and not abrogated by re-feeding mice after APAP injection (Supplementary Fig. S8E). Liver bulk RNA sequencing confirmed, next to several catabolic processes, a strong induction of oxidative phosphorylation, autophagy and oxidative stress responses (Fig. 6H, Supplementary Fig. S8F, G). Fasting provoked mitochondrial fusion indicated by upregulation of fusion and downregulation of fission genes (Fig. 6H, right), which presumably promotes more efficient mitochondrial energy production despite glucose deprivation. Although this concomitantly increases ROS production, fasting also led to pronounced antioxidant- and autophagy/mitophagy-related gene expression (Fig. 6H, right). Hence, fasting prepares the cells for APAP-induced low energy, high ROS conditions by previously inducing these processes that are well-established to be protective against APAP toxicity [32–34]. Of note, all key events in APAP- and other metabolism-targeting agents-induced liver damage, including catabolic processes, autophagy, mitochondrial morphology, cellular respiration, and oxidative stress, are strongly interconnected and strikingly involve the contribution of several BCL-2 proteins (Supplementary Fig. S9). This intertwined net proposes non-apoptotic roles of BCL-2 proteins in regulating general stress responses that thus may also modulate necrotic forms of cell death.

### Increased glycolysis allows fasting-initiated autophagy to remove damaged mitochondria

**Fig. 7:**
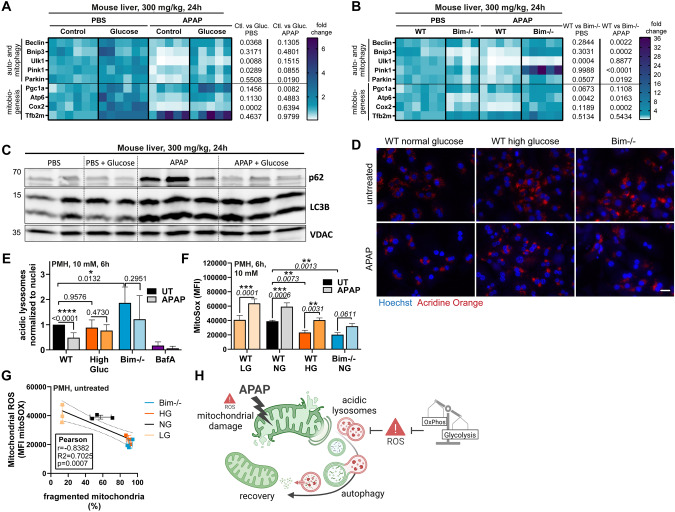
Increased glycolysis allows fasting-initiated autophagy to remove damaged mitochondria. **A**–**B** Transcript levels of livers of mice treated with 300 mg/kg APAP for 24 h with previous glucose injection (**A**) or *Bim*−/− mice (**B**). Data shows fold change to WT control/PBS and respective statistical significances on the right. **C** Western Blot of liver lysates of mice injected with 300 mg/kg APAP for 24 h with previous glucose injection. **D**–**E** Representative immunofluorescence images (**D**) and quantification (**E**) of Acridine Orange and Hoechst-stained primary murine hepatocytes (PMH) cultured in normal or high glucose (High Gluc) and untreated (UT) or treated with 10 mM APAP or Bafilomycin (BafA) for 6 h, *n* = 4–7. Scale bars 100 µm. Bright Acridine Orange signals were normalized to Hoechst signals. **F** Quantification of median fluorescence intensity (MFI) of MitoSox Red-stained PMH cultured in low (LG), normal (NG), or high glucose (HG) and treated with 10 mM APAP for 6 h, *n* = 3. **G** Correlation between MitoSox Red MFI and fragmented mitochondria calculated from length to width ratio of MitoTracker Green-stained untreated WT/*Bim−/−* PMH cultured in LG, NG, or HG. SD is shown of the displayed *n* = 3. Statistical significance and linear fit calculated from Pearson correlation. **H** Schematic illustration of how reduced mitochondrial dependency circumvents ROS-mediated inhibition of mito/autophagy in response to APAP treatment. Data points and/or bar graphs are mean +/− SD with *n* as independent biological replicates. Statistical significance was tested using Two-way ANOVA with Sidak’s multiple comparison test (**A**, **B**, **E**, **F**).

Since induction of auto- and mitophagy seem to be decisive for the reduced pathology of fasted APAP-treated mice, we next aimed to understand how BIM deficiency or glucose administration, both promoting a glycolytic energy profile, affect these processes. APAP treatment of not glucose-treated wild type mice caused a pronounced downregulation of autophagy genes after 24 h compared to PBS (Fig. 7A, B). In contrast, BIM-deficient or glucose-treated mice showed elevated autophagy and mitochondrial biogenesis gene expression compared to APAP-treated control mice (Fig. 7A, B, APAP). It must be noted that APAP-treated wild type mice experienced more severe liver damage compared to the other conditions (Figs. 2 and 5) implicating that those liver samples contain more dead/dying cells. Dying cells presumably decrease gene expression. However, as we used similar levels of cDNA for quantitative RT-PCR and further normalized the resulting data to β-actin mRNA, it is reasonable to draw conclusions on relative changes. Although APAP treatment resulted in lipidation of LC3B protein, a hallmark of ongoing autophagy, simultaneous p62 accumulation suggested a blocked autophagic flux in wild type livers (Fig. 7C). In contrast, in glucose-treated mice, p62 did not accumulate and LC3B was processed, indicating successful execution of autophagy and removal of damaged organelles (Fig. 7C). Bafilomycin A co-treatment resulted in p62 accumulation in glucose- and APAP-treated hepatocytes, confirming ongoing autophagy in glycolytic cells (Supplementary Fig. S10A). Autophagic flux describes that autophagic vesicles fuse with lysosomes, in which the disassembly of engulfed components takes place. It is believed that smaller lysosomes are less acidic and rather catabolically inactive [35]. Accordingly, total numbers and size of remaining lysosomes were reduced upon APAP treatment, which was considerably less pronounced in BIM-deficient or high glucose-conditioned hepatocytes (Fig. 7D, E). Interestingly, BIM deficiency went along with an in general increased lysosome abundancy (Fig. 7D, E). Lysotracker and Cathepsin B staining confirmed these findings (Supplementary Fig. S10B). Low oxidative stress is required for lysosomal acidity and functionality of lysosomal proteases. And remarkably, hepatocytes with a glycolytic profile showed significantly reduced steady-state mitochondrial ROS production (Fig. 7F), explaining their lysosomal phenotype and their ability to finish autophagy. Although oxidative stress increased after APAP treatment in all conditions, it was substantially delayed in glucose-conditioned hepatocytes and accelerated in starved cells (Supplementary Fig. S10C). This was confirmed by GSH measurement in primary hepatocytes and mitochondrial ROS measurement in HepG2 cells (Supplementary Fig. S10D, E). Importantly, reduced mitochondrial ROS production strongly correlated with mitochondrial fission, i.e. lower dependency on mitochondria as energy source (Fig. 7G). Notably, the beneficial effects of increased glycolysis on APAP-induced ROS production were also observed at low oxygen levels, i.e. under a more physiological condition (Supplementary Fig. S10F). Hence, glycolytic cells may be damaged by APAP likewise as mitochondria-dependent cells, however, due to their lower ROS levels, damaged mitochondria can be recycled via lysosomal autophagy and cells can recover (Fig. 7H). This line of argumentation is supported by Song and Hwang et al. that demonstrated that glucose-deprived cells are in general less capable of autophagy execution [36]. The autophagic flux is blocked due to elevated ROS resulting from higher oxidative phosphorylation rates preventing lysosomal acidification [36]. Worth mentioning, BIM-deficient hepatocytes show similarly low ROS levels than glucose-conditioned cells (Fig. 7G), although BIM deficiency also resulted in higher oxidative phosphorylation activity (Fig. 3), suggesting an additional mechanism how BIM regulates redox homeostasis. In summary, our study reveals that nutritional conditions critically determine AILI severity. Preceding caloric restriction initiates autophagy induction, which is further induced upon APAP intoxication. However, autophagy can only be executed in cells with a predominant glycolytic profile as they have reduced ROS and can maintain lysosomal function.

## Discussion

Drug-induced liver toxicity is one of the severe side-effects of various therapeutic drugs, with AILI as the most frequent and most fatal example. Despite the long history of AILI research, intracellular processes, in particular how hepatocytes ultimately die, are still not fully understood and controversial. Several studies claimed certain compounds to serve as antidotes based on a reduction in apoptotic markers [11], which led to misleading conclusions and overall slowed down effective therapy development. Thus, understanding the paradoxical cell death events induced by APAP intoxication is pivotal for new therapeutic approaches.

Our present study clarifies how BIM promotes necrotic cell death by providing strong evidence for a novel non-canonical, non-apoptotic function of BIM in the regulation of mitochondrial morphology and energy metabolism. Even in untreated cells BIM deletion resulted in fragmented mitochondria and increased glycolysis, which protected hepatocytes from APAP-induced mitochondrial damage, associated drop in cellular ATP levels, ROS generation and ultimately from undergoing oncotic necrosis. Astonishingly, simple glucose administration mimicked the glycolytic phenotype of BIM-deficient hepatocytes, and consequently also protected hepatocytes and mice from AILI. Thus, we here reveal a general rule that hepatocytes’ bioenergetic profile determines the outcome of AILI with predominantly cells dependent on mitochondrial ATP generation being sensitive to APAP toxicity. Increased glycolytic energy production is beneficial as it permits to maintain sufficiently high ATP and low ROS levels during mitochondrial damage and malfunction. This enables the removal of damaged and ROS-producing mitochondria by mitophagy, overall resulting in significant protection of mice from APAP-induced liver damage.

The superior ATP levels in BIM-deficient cells were attributed to both, increase in oxidative phosphorylation and in glycolysis. BIM-deficient hepatocytes and cell lines displayed pronounced mitochondrial fragmentation, a hallmark of increased glycolysis [24]. The correlation between mitochondrial morphology and metabolic profile was further underpinned in MFN1/2-deficient cells, resulting in fission and an increased glycolytic to mitochondrial ATP ratio, which ultimately rescued from APAP-induced cell death. Interestingly, it had been previously reported that BIM deletion in mice affects body weight, fat deposition and the abundancy of some energy metabolites [37]. More efficient glucose uptake and the elevated levels of Hexokinase-I in *Bim*−/− cells and mice possibly explain the increased contribution of glycolysis to total ATP generation in these cells. It has been furthermore demonstrated that BIM interacts with Hexokinases at mitochondria and that they mutually modulate their activities [38–40]. Interestingly, BAX and BAK can also modulate mitochondrial morphology by interaction with Mitofusins and DRP1, which was demonstrated to be partly independent of cell death induction [26–28]. As we did not find that BIM interacts with Mitofusins or DRP1, and that BAX/BAK deficiency does not elevate expression of Hexokinases, we speculate that the mitochondrial fragmentation in BIM-deficient cells is caused by other mechanisms than in BAX/BAK-deficient cells. Likely, BIM deletion affects primarily glycolysis, e.g. via upregulating glucose uptake, and Hexokinases expression and activity, and that the observed mitochondrial fragmentation happens as a secondary consequence. In line of this notion is our observation that glucose administration to wild type hepatocytes also results a shift towards glycolysis and associated mitochondrial fragmentation. In summary, the distinct phenotype of untreated BIM-deficient cells, the protection by glucose administration or by Mitofusin deletion (which both mimics the BIM−/− phenotype), all strongly supports the idea of a MOMP-unrelated function of BIM. Notably, although a number of BIM-deficient mice die before birth it is rather unlikely that unspecific adaptations of the surviving mice facilitate phenotype and the protection against APAP because both CRISPR/Cas9-generated BIM-deficient cell lines recapitulated the observed effects.

A critical question remains how BIM regulates mitochondrial ATP production. Most likely, this activity of BIM is related to its interactions with anti-apoptotic members of the BCL-2 family on the mitochondrial membrane. In contrast to the yet unexplored non-canonical functions of BH3-only proteins, BCL-2, BCL-x_L_ and MCL-1 have been reported to regulate mitochondrial membrane potential [41, 42], fission and fusion [43–45], oxidative phosphorylation [44, 46–48], as well as mitophagy [49]. BIM interacts with high affinity with all of these anti-apoptotic BCL-2 homologs leading to their sequestration [50], and may hereby impact also their non-canonical activity on mitochondrial respiration. BCL-2 was shown to bind and hyperactivate complex IV, while BCL-x_L_ and MCL-1 positively regulate complex V assembly and function, notably in a BH2 and c-terminal domain-dependent manner [43–48]. Thus, mitochondrial ATP production in BIM-deficient cells is presumably elevated by stabilized interaction between respiratory complexes and non-sequestered, “free” BCL-2-like proteins. In addition, the low mitochondrial ROS levels observed in BIM-deficient cells, which decisively contribute to protection from AILI, are likely also the result of non-sequestered BCL-2 [51, 52]. The interaction of BIM with anti-apoptotic BCL-2 homologs is especially critical when stress signals promote BIM expression, disturbing the balance between anti- and pro-apoptotic BCL-2 proteins, thereby favoring apoptosis. However, BIM is also expressed at high levels under non-lethal conditions indicating non-canonical functions under steady-state conditions [53, 54]. Furthermore, cellular activation promotes increased BIM expression in the absence of cell death induction, as for example observed in lipopolysaccharide (LPS)-activated neutrophilic granulocytes and lectin-activated T lymphocytes [55]. Interestingly, LPS or bacterial infection are known to prime neutrophils for ROS production [56], and it is tempting to speculate that LPS-induced BIM upregulation may relevantly contribute to this process, as we here show that BIM deficiency results in lower ROS levels.

BID- and PUMA-deficient mice have also been reported to be protected from AILI [19, 20]. Interestingly, here only BIM deletion appeared to be protective against AILI, whereas deletion of NOXA did not result in reduced liver necrosis, but if anything enhanced it. BIM, BID and PUMA can bind to all anti-apoptotic BCL-2 family members with high affinity, while NOXA only interacts with MCL-1 [50], presumably minimizing the impact of NOXA deletion on the bioenergetic profile. However, it remains to be investigated whether BID and PUMA regulate mitochondrial functions and associated energy metabolism like BIM. In contrast to BIM deficiency, their deletion does not result in reduced body weight or fat content, highlighting a potentially unique role of BIM in regulating energy metabolism. Future studies must clarify whether BIM regulates these processes directly, or as discussed here indirectly via BCL-2-like proteins.

APAP-induced liver necrosis occurs predominantly around the hepatic central vein. This can be explained by high local CYP2E1 expression (converting APAP to NAPQI), however, as our data points out, also by the nutrient- and oxygen-poor environment, which causes centrilobular hepatocytes to be generally more susceptible to ATP-depleting substances. Due to the oxygen-poor environment, centrilobular hepatocytes adapted to a relatively high glycolytic capacity, but they cannot push the limits as glucose is limited. Thus, systemically increasing glucose levels likely elevates also the centrilobular glucose concentration, where the resident hepatocytes can increase their glycolysis rate resulting in attenuated sensitivity to ATP-depleting substances. In addition to metabolic abundancy, spatial protein analysis could possibly reveal distinct expression and interaction patterns of BCL-2 proteins that correlate with the specific, zonation-dependent metabolic tasks of hepatocytes [57] or even other body’s nutrient axes.

A last important aspect of our study that deserves further attention is the effect of caloric restriction on AILI. Ever since, it was suggested that fasting is required for APAP to induce hepatocyte death and liver damage. Here, we show for the first time that fasted mice are surprisingly less sensitive to APAP than non-fasted mice, as monitored by longer survival and attenuated hemorrhage. At first glance this may appear contradictory to our finding that glucose administration protects from AILI. However, caloric restriction resulted in an AMPK-regulated increase in catabolic processes, such as the turnover of mitochondria. AMPK activation and mitophagy are well-established to reduce the severity of APAP-induced mitochondrial damage due to lower ROS levels [58–62]. Paradoxically, starvation may therefore have a similar effect than glucose administration, i.e., it reduces the impact of mitochondria-dependent cellular energy production and ROS formation during APAP intoxication. Thus, overall, combination of fasting and glucose administration result in reduced damage and at the same time permit the removal of damaged mitochondria by mitophagy.

Our study also suggests that targeting BIM in cancer therapy may initiate two independent processes with potential synergies in the induction of cancer cell death. Classical chemotherapeutic drugs may result in BIM stabilization and activation, promoting in turn BAX and BAK activation, MOMP and apoptosis induction. Simultaneous inhibition of glycolytic ATP production and increased ROS generation by elevated BIM levels may render tumor cells, often showing a more glycolytic phenotype, increasingly dependent on mitochondrial oxidative phosphorylation, and thus more susceptible to mitotoxicants. A potential shift from classical apoptosis to a more necrotic mode of cell death may in turn result in immunogenic cell death with enhanced anti-tumor immune responses [63]. Thus, the here described novel non-canonical function of BIM may have wide-spread impact, not only on drug-induced organ damage, but also on the efficacy of cancer therapy.

In summary, our study reports a so far undescribed non-canonical, apoptosis-independent role of BIM in the regulation of mitochondrial dynamics and energy metabolism, which critically contributes to APAP-induced necrotic liver damage. This likely applies to several other necrotic hepatic diseases involving BCL-2 family members, such as Thioacetamide intoxication or ischemia/reperfusion injury. In addition to unraveling paradoxical cell death events during AILI, we provided strong evidence that the hepatotoxicity of APAP critically depends on the cellular bioenergetic profile, and that simple glucose administration protects from AILI. Hepatocytes are extremely rich in mitochondria due to their high energy demand, but incoming threats often affect mitochondrial health and energy production causing hepatocyte intoxication. Oxidative stress, mostly derived from damaged or highly active mitochondria, represents the most devastating factor in AILI as it is cause and consequence in the vicious cycle of APAP-induced mitochondrial damage. Decreasing the dependency on mitochondria permits to escape from this loop and is therefore key to prevent AILI. Our findings furthermore clarify conflicting results in AILI research and contribute to a more detailed understanding of interconnected processes regarding cellular metabolism and oxidative stress, providing a basis for the development of novel therapeutic approaches for patients with APAP intoxication and other pathologies.

## Methods and materials

For detailed methods and materials please see Supplementary File.

For uncropped Western blots see Supplementary Material.

### Supplementary information

Suppl. Material
