# Supplementary material for: Non-canonical BIM-regulated energy metabolism determines drug-induced liver necrosis
Source: Cell Death Differ. 2023 Nov 24;31(1):119–31. doi: 10.1038/s41418-023-01245-7 (PMC10781779; doi:10.1038/s41418-023-01245-7)
Supplement: Supplementary file 1 — Suppl. Material [file 41418_2023_1245_MOESM1_ESM.pdf]

SUPPORTING INFORMATION

Title: Non-canonical BIM-regulated energy metabolism determines drug-induced liver necrosis.

Authors:

Rebekka Lambrecht, Franziska Rudolf, Anna-Katharina Ückert, Valentina C. Sladky, Truong San Phan, Jasmin Jansen, Samara Naim, Thomas Kaufmann, Adrian Keogh, Susanne Kirschnek, Aswin Mangerich, Florian Stengel, Marcel Leist, Andreas Villunger, Thomas Brunner[#]

#: Corresponding author, thomas.brunner@uni-konstanz.de

Content

SUPPLEMENTAL FIGURES 2
SUPPORTING METHODS 16
SUPPORTING MATERIAL 30
REFERENCES 37

List of Tables

Supplementary Table 1: Mice. 30
Supplementary Table 2: Cell lines. 30
Supplementary Table 3: Oligonucleotides for guide RNA for CRISPR/Cas9 Knockout Generation. 30
Supplementary Table 4: Oligonucleotides for forward (fw) and reverse (rev) primers for RT-qPCR. 30
Supplementary Table 5: Primary and secondary antibodies for Western Blot and Immunoprecipitation. 32
Supplementary Table 6: Plasmids. 33
Supplementary Table 7: Software. 34
Supplementary Table 8: Chemicals/Drugs. 34
Supplementary Table 9: Commercial Kits/Assays. 36
Supplementary Table 10: Technical Devices. 36

SUPPLEMENTAL FIGURES

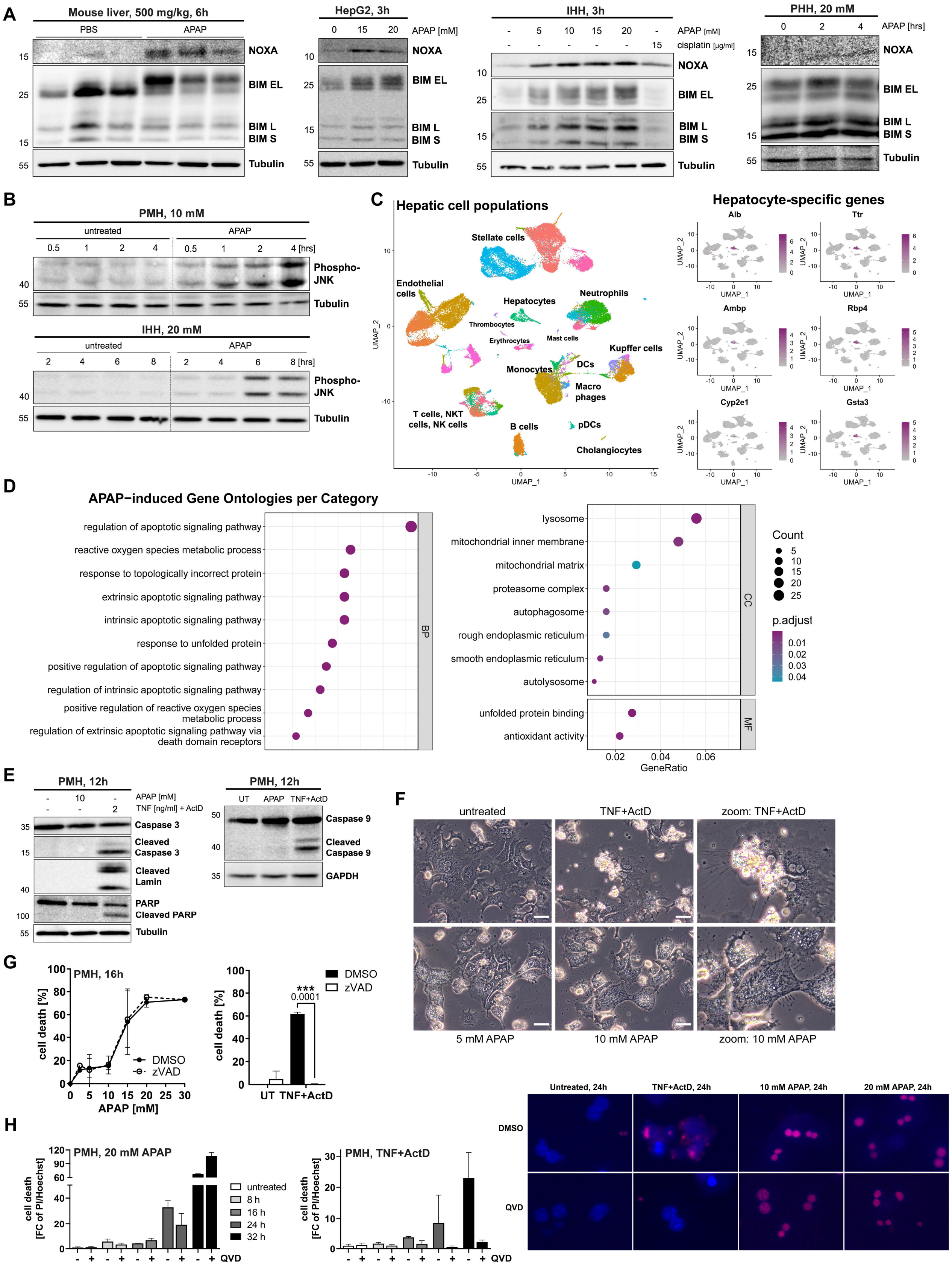

Fig. S1: Induction of BH3-only proteins in APAP-provoked necrosis, related to Fig. 1.

A Western Blot of mouse liver (500 mg/kg APAP, 6h), HepG2 and IHH treated for 3 h with APAP or cisplatin and primary human hepatocytes (PHH) treated with 20 mM APAP.

B Western Blot of primary murine hepatocytes (PMH) and IHH treated with 10 mM (PMH) or 20 mM APAP (IHH).

C UMAP plots showing hepatic cell populations from single cell RNA sequencing of mice treated for 6 h with PBS or 500 mg/kg APAP. Cells are colored based on annotation taken from Kolodziejczyk *et al.* [1]. Hepatocyte-specific genes confirm hepatocyte cluster (right).

D Selected gene ontologies from Top 100 GOs of hepatocyte subset from single cell RNA sequencing data described in (C).

E Western Blot of PMH treated with APAP or murine TNF and 30 nM ActD for 12 h.

F Representative phase contrast microscopy of PMH treated with APAP or 2 ng/ml murine TNF and 30 nM ActD for 12 h. Scale bar shows 50 µm.

G MTT assay of PMH (16h) treated in combination with 30 µM z-VAD-fmk or DMSO; UT: untreated. n=3.

H Quantification of PI-Hoechst stained PMH treated with 20 mM APAP or 2 ng/ml murine TNF and 30 nM ActD for different timepoints. Data show mean +/- SD, n=3

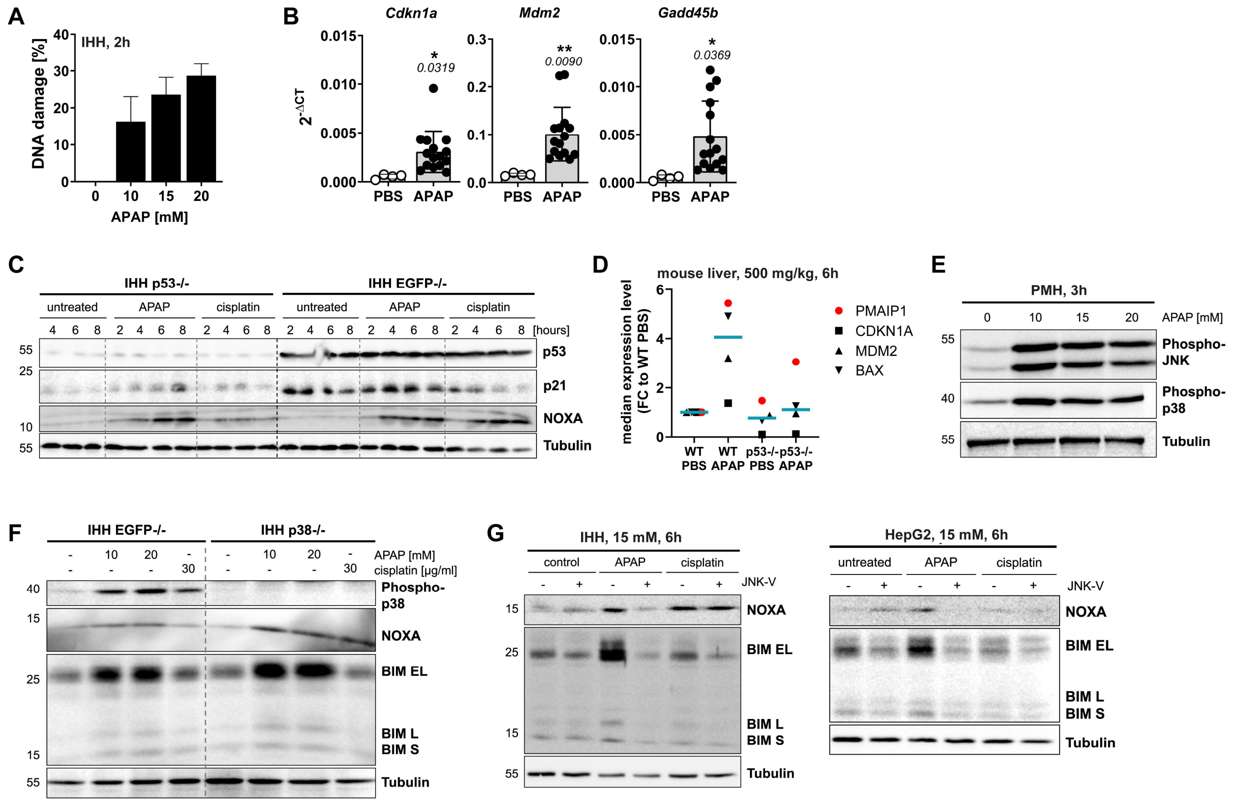

Fig. S2: NOXA is induced via p53 and JNK after APAP treatment, related to Fig. 2.

A FADU assay of primary murine hepatocytes (PMH) treated for 2 h APAP, n=4.

B Transcript levels of mouse livers treated for 6 h with 500 mg/kg APAP i.p.

C Western Blot of p53-deficient or control IHH cells treated with 20 mM APAP or 30 µg/ml cisplatin.

D Transcript levels of WT or p53-/- mouse livers, n=3 as fold change to WT PBS. Blue line indicates median of all genes.

E Western Blot of PMH treated with APAP for 3 h.

F Western Blot of p38-deficient or control IHH cells treated with APAP or cisplatin for 4 h.

G Western Blot of IHH and HepG2 cells treated with 15 mM APAP or 15 µg/ml cisplatin for 6 h and 30 min pre-treatment with 4 µM JNK-V or DMSO.

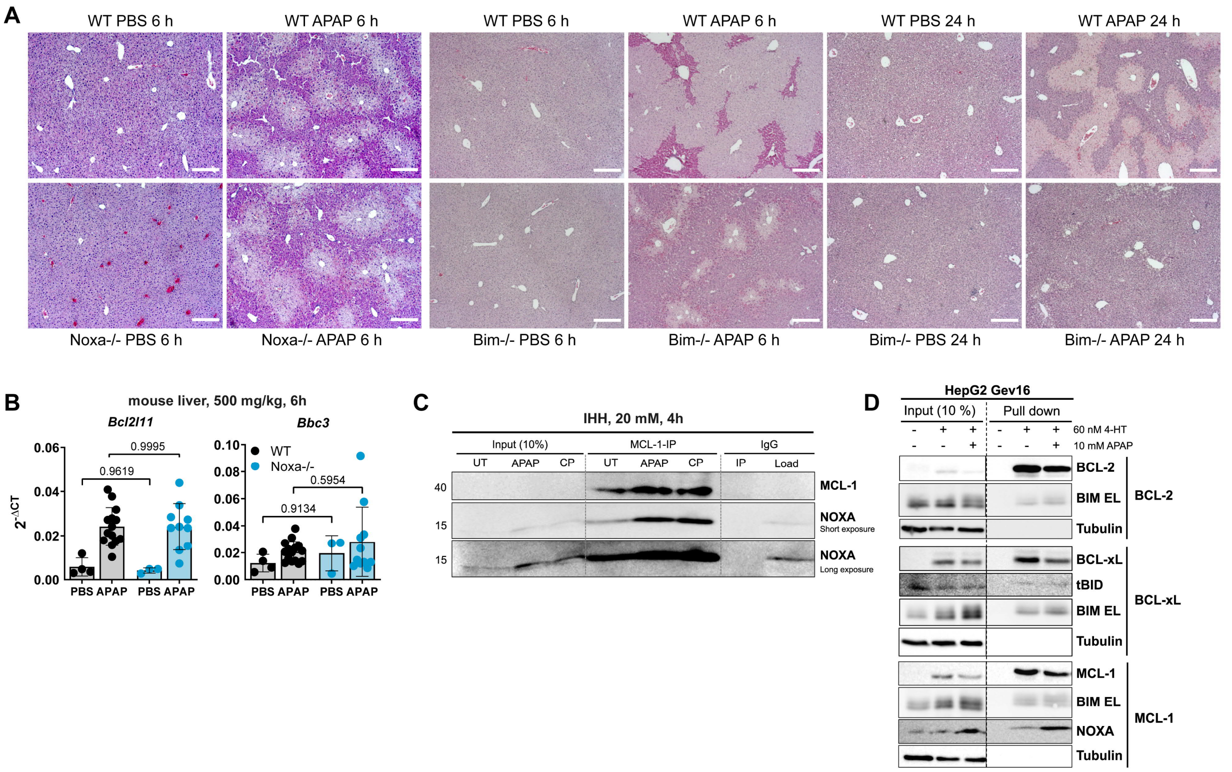

Fig. S3: BIM and NOXA contributes to BCL-2 interactome during early APAP intoxication, related to Fig. 2.

A Representative bright-field images of H&E-stained liver sections from WT, *Noxa-/-*, *Bim-/-* mice treated with either PBS, 500 mg/kg APAP for 6 h or 300 mg/kg APAP for 24 h. Scale bars 250 µm.

B Transcript levels of livers of WT and *Noxa-/-* mice.

C Western Blot of MCL-1 immunoprecipitation of IHH untreated (UT) or treated for 4 h with 20 mM APAP or 30 µg/ml cisplatin (CP).

D Western Blot of respective immunoprecipitations of HepG2 Gev16 cells that 4HT-inducibly express BCL-2, BCL-xl or MCL-1 treated as indicated for 4 h. Protein names on the right show respective overexpression and immunoprecipitation.

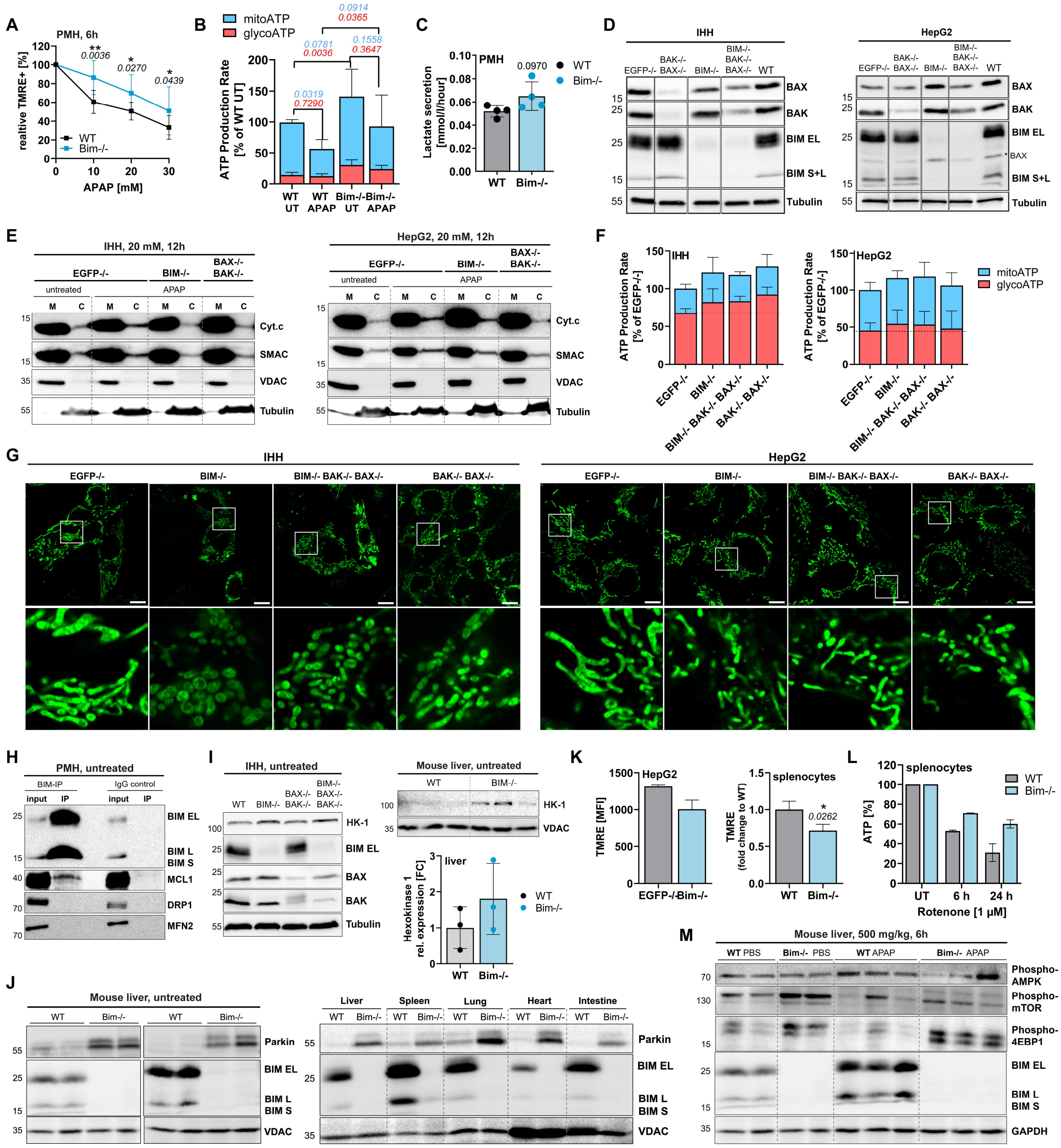

Fig. S4: BIM deficiency is accompanied with enhanced ATP production and fragmented mitochondrial morphology, related to Fig. 3.

A Live, MitoTracker Green+, TMRE+ primary murine hepatocytes (PMH) treated for 6h, n=4.

B related to Figure 3A: Seahorse Induced ATP Rate Assay with medium (UT) or APAP injection (final 15 mM) of primary murine hepatocytes (PMH) from WT and *Bim-/-* mice, n=4-8. Statistical significance was tested by comparing mitoATP and glycoATP independently.

C Lactate production of primary murine hepatocytes (PMH) measured with LAC143 Kit over time, n=4.

D Western Blot of untreated HepG2 and IHH to verify deletion of BIM, BAX and BAK. *EGFP*-/- cells serve as control cell line and were generated with guideRNAs against *EGFP* gene.

E Western Blot of mitochondrial (M) and cytosolic (C) fractions of the different IHH and HepG2 cell lines treated for 12 h with 20 mM APAP.

F Bioenergetic profile of the different IHH and HepG2 cell lines determined by Seahorse ATP Rate Assay, n=4-7.

G Representative confocal microscopy images of Mitotracker Green-stained IHH and HepG2 cell lines, n=4. Scalebar 20 µm.

H Western Blot of BIM immunoprecipitation and IgG control of untreated PMH.

I Western Blot for Hexokinase 1 of untreated IHH cells and whole liver lysates from untreated mice, n=3, with quantification relative to VDAC signal below.

J Western Blot of liver and tissue lysates of untreated WT and *Bim*-/- mice.

K Flow cytometry analysis of TMRE median fluorescence intensity (MFI) of live, MitoTracker Green positive HepG2 cells or splenocytes from WT and *Bim*-/- mice, n=2 (HepG2) or n=3 (splenocytes).

L CellTiter-Glo Assay of splenocytes from WT and *Bim*-/- mice, untreated (UT) or treated with Rotenone, n=2.

M Western Blot of whole liver lysates of mice treated with 500 mg/kg APAP for 6h.

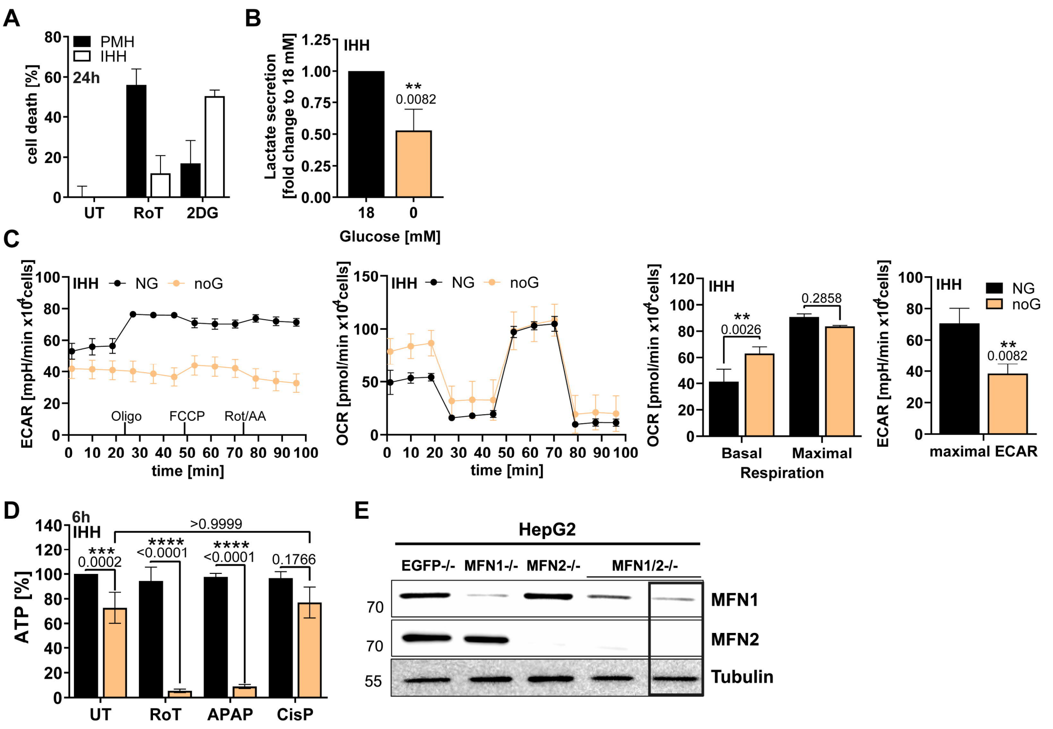

Fig. S5: Glycolytic IHH cells can be sensitized to APAP by depriving glucose, related to Fig. 4.

A MTT assay of primary murine hepatocytes (PMH) and IHH untreated (UT) or treated with 0.5 µM Rotenone (RoT) or 9 mM 2-Deoxyglucose (2-DG) for 24 h, n=4.

B LAC142 Assay of IHH cultured in normal or no glucose containing media, n=3.

C related to Figure 4E (IHH): Seahorse Mito Stress Test Assay extracellular acidification rate (ECAR) and oxygen consumption rate (OCR) time course with Oligomycin (Oligo) injection at 15 min, FCCP at 45 min and Rotenone + Antimycin A(Rot/AA) at 80 min with calculated respiration and maximal ECAR, n=3.

D CellTiter-Glo Assay of IHH treated for 6 h. n=3.

E Western Blot of untreated HepG2 cells to verify deletion of MFN1 and MFN2. Black rectangle indicates the MFN1/2-/- cell line used for experiments shown in Figure 4.

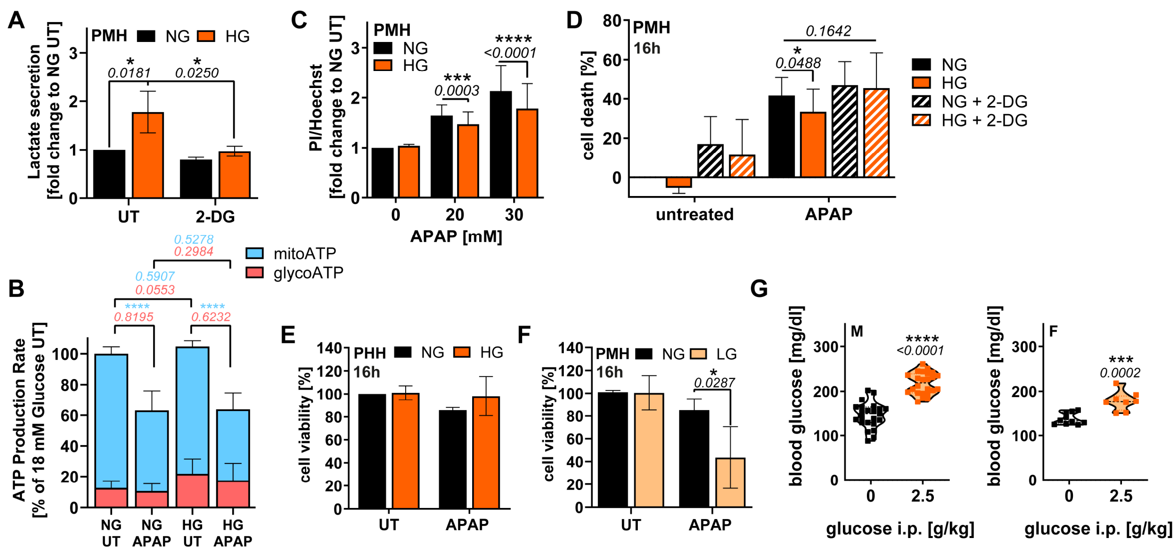

Fig. S6: Caspase-independent cell death in glycolytic, APAP-intoxicated cells and mice, related to Fig. 5.

A LAC134 Assay of primary murine hepatocytes (PMH) cultured in normal glucose (NG) or high glucose (HG), untreated (UT) or treated with 50 mM 2-Deoxyglucose, n=3.

B related to Figure 5A: Seahorse Induced ATP Rate Assay with medium (untreated, UT) or APAP injection (final 15 mM) of primary murine hepatocytes (PMH) from WT and *Bim-/-* mice, n=3-8. Statistical significance was tested by comparing mitoATP and glycoATP independently.

C PI / Hoechst fluorescence ratio of PMH cultured in normal (NG) or high glucose (HG) media treated with APAP for 16 h, n=4.

D MTT assay of PMH cultured in normal (NG) or high glucose (HG) media and treated with 10 mM APAP and 50 mM 2-Deoxyglucose for 16 h, n=5.

E MTT assay of primary human hepatocytes (PHH) cultured in NG or HG and treated (24h), n=2.

F MTT assay of PMH cultured in (NG) or low glucose (LG) and treated (16h), n=3.

G Blood glucose levels measured of male (M) and female (F) mice 1 h after i.p. injection of 2.5 g/kg glucose. Bold dotted line indicates median and weak dotted lines show 25% and 75% quartiles.

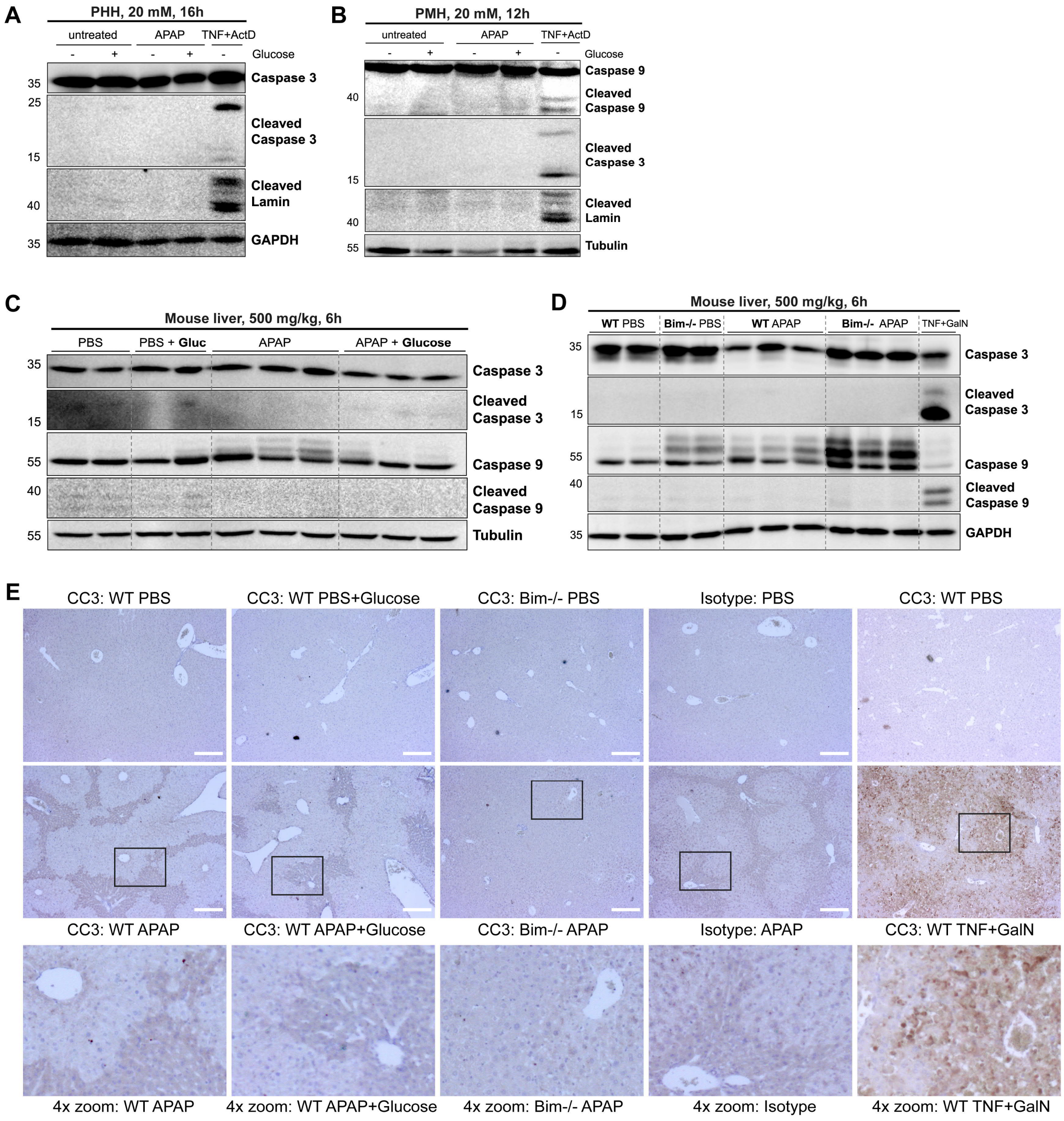

Fig. S7: Caspase-independent cell death in glycolytic, APAP-intoxicated cells and mice, related to Fig. 5.

A-B Western Blot of primary human and murine hepatocytes (PHH/PMH) cultured in normal (-) or high glucose (+) media and treated for 16 h (PHH) or 12 h (PMH) with 20 mM APAP or 1 ng/ml human or murine TNF + 30 nM ActD.

C-D Western Blot liver lysate from WT and *Bim*-/- mice treated with 500 mg/kg APAP or 25 µg/kg TNF and 1 g/kg N-galactosamine (GalN) for 6 h with or without injection of 2.5 g/kg glucose (Gluc) 1 h prior to APAP.

E Representative immunohistochemistry images of anti-cleaved Caspase-3-stained liver sections from mice treated for 6 h with 500 mg/kg APAP with optional injection of 2.5 g/kg glucose 1 h prior to APAP. As positive control liver from mice treated with 25 µg/kg murine TNF and 1 g/kg N-galactosamine (GalN) were used. Scale bars 250 µm.

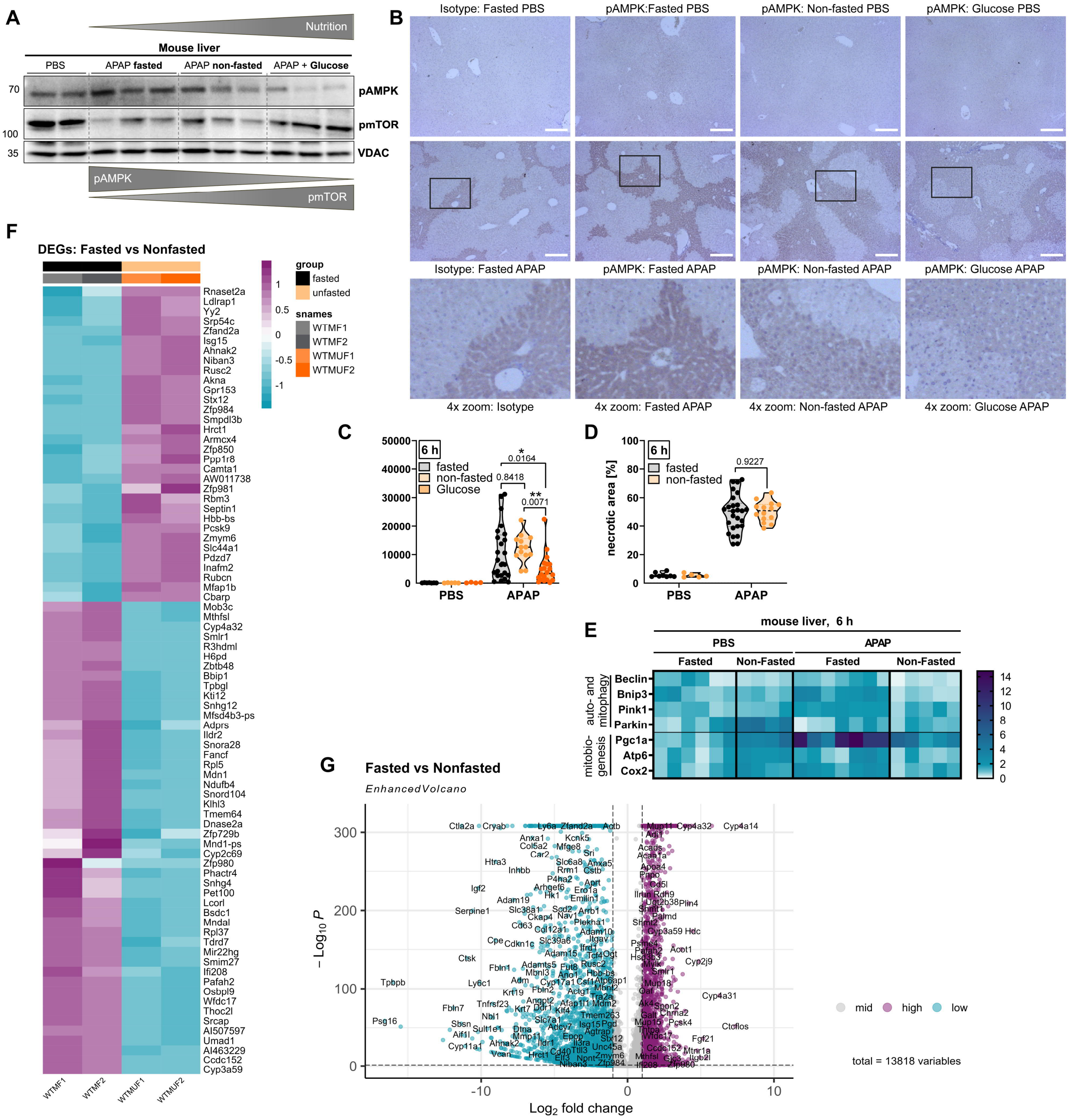

Fig. S8: Fasting induces AMPK activation and cellular catabolism, related to Fig. 6.

A Western Blot of liver lysates of nonfasted, fasted, or glucose injected mice treated with APAP (500 mg/kg APAP, 6h).

B Representative immunohistochemistry images of anti-phospho-AMPK-stained liver sections from non-fasted, fasted, or additional 2.5 g/kg glucose-treated mice (500 mg/kg APAP, 6h). Scale bars show 250 µm.

C-D Serum ALT levels and necrotic area determined from H&E-stained liver sections of non-fasted, fasted, or additional 2.5 g/kg glucose-treated mice (500 mg/kg APAP, 6h). Bold dotted line indicates median and weak dotted lines show 25% and 75% quartiles. Statistical significance was tested using two-way ANOVA with Sidak’s multiple comparison test.

E Mice were either left with food or fasted overnight prior to injection with 500 mg/kg APAP for 6 h. RNA isolated from livers were analyzed by quantitative reverse transcriptase-PCR. Data shows fold change to non-fasted PBS samples.

F-G Volcano plot and heatmap of top fasting-induced differentially expressed genes (DEGs) from liver bulkRNA sequencing from fasted and non-fasted mice, WTMF = WT male fasted mouse, WTMUF = WT male unfasted mouse.

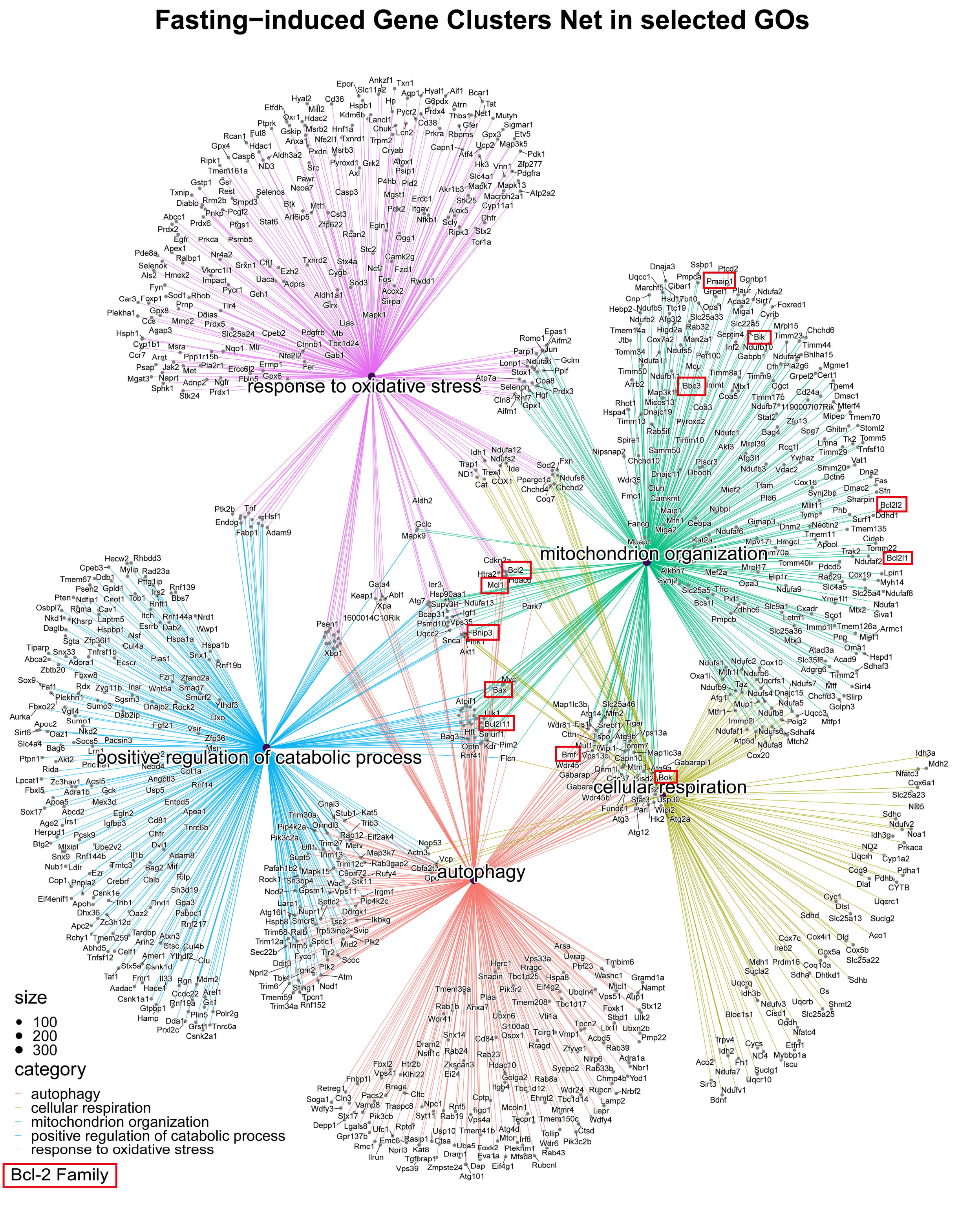

Fig. S9: BCL-2 family members are involved in fasting-induced processes independent of apoptosis, related to Fig. 6.

Cluster Map of selected fasting-induced gene ontology pathways with respective DEGs from liver bulkRNA sequencing. BCL-2 family proteins are highlighted in red.

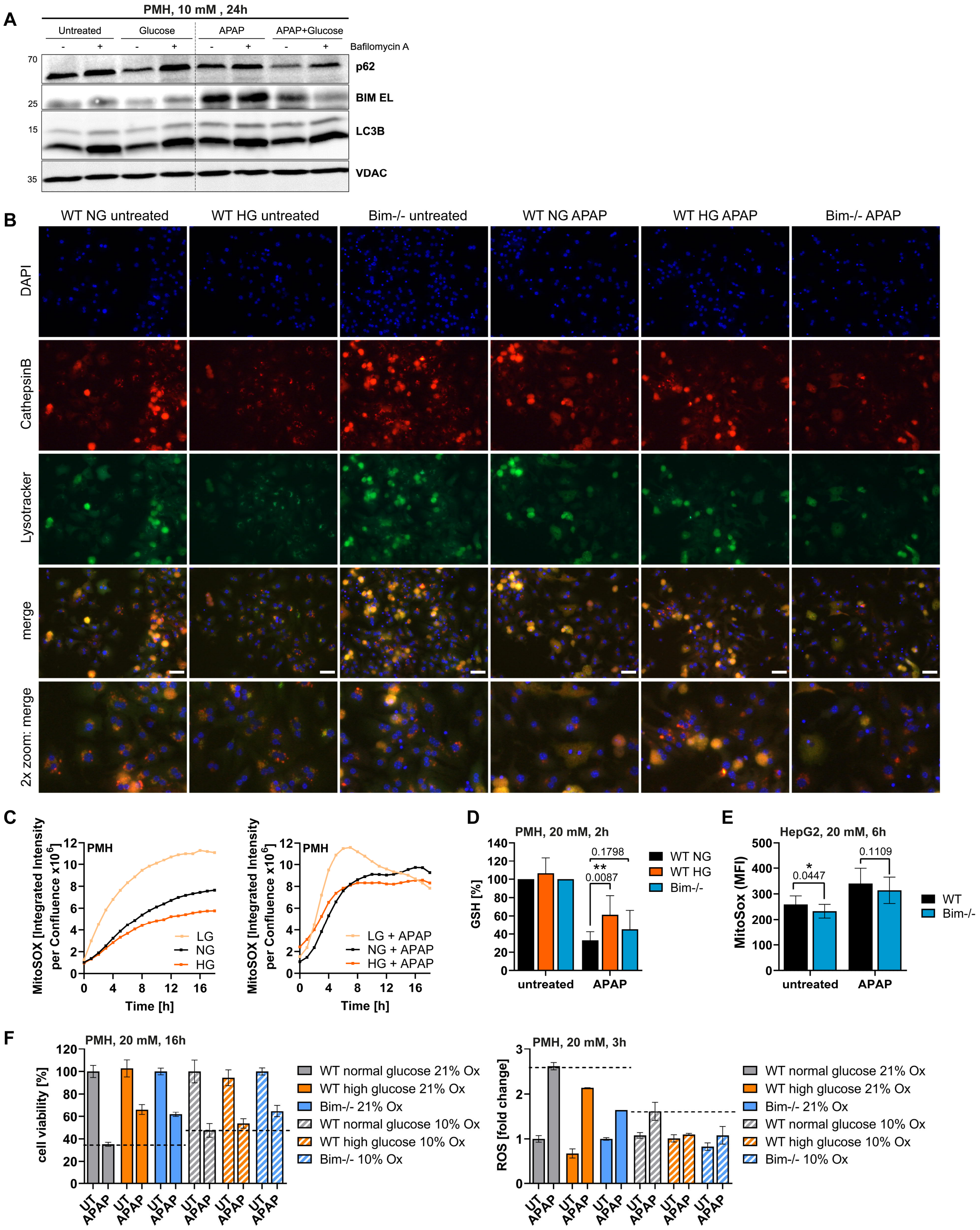
A
PMH, 10 mM , 24h
Untreated
Glucose
APAP
APAP+Glucose
Bafilomycin A
p62
BIM EL
LC3B
VDAC
B
WT NG untreated
WT HG untreated
Bim-/- untreated
WT NG APAP
WT HG APAP
Bim-/- APAP
DAPI
CathepsinB
Lysotracker
merge
2x zoom: merge
C
MitoSOX [Integrated Intensity per Confluence x10^6]
PMH
LG
NG
HG
LG + APAP
NG + APAP
HG + APAP
Time [h]
D
PMH, 20 mM, 2h
GSH [%]
WT NG
WT HG
Bim-/-
0.1798
0.0087
untreated
APAP
E
HepG2, 20 mM, 6h
MitoSox (MFI)
0.0447
0.1109
WT
Bim-/-
untreated
APAP
F
PMH, 20 mM, 16h
cell viability [%]
PMH, 20 mM, 3h
ROS [fold change]
WT normal glucose 21% Ox
WT high glucose 21% Ox
Bim-/- 21% Ox
WT normal glucose 10% Ox
WT high glucose 10% Ox
Bim-/- 10% Ox
UT
APAP

Fig. S10: Glucose administration and BIM deficiency regulate autophagy, lysosomes, and mitochondrial ROS levels, related to Fig. 7

A Western Blot analysis of primary murine hepatocytes (PMH) cultured in normal or high glucose media, treated for 24 h with 10 mM APAP with additional 100 nM Bafilomycin A treatment 2 h prior to harvesting.

B Representative immunofluorescence images of Hoechst, LysoTracker Green and Cathepsin Magic Red-stained WT and *Bim*-/- PMH cultured in normal (NG) or high glucose (HG) media and treated for 6 h with 10 mM APAP. Scale bars show 100 µm.

C Quantification of fluorescence intensity of MitoSOX Red-stained PMH overtime cultured in low (LG), normal (NG), or high glucose (HG) media and treated with 10 mM APAP directly prior to measurement with Incucyte. Data points show representative single biological replicate of two independent experiments.

D Quantification of luminescence from GSH/GSSG-Glo Assay of WT or *Bim*-/- PMH cultured in normal (NG) or high glucose (HG) media treated for 2 h with 20 mM APAP, n=3-8.

E Flow cytometry median fluorescence intensity (MFI) of live MitoSox Red-stained HepG2 cells treated for 6 h with 20 mM APAP, n=3.

F (PMH) were isolated and after adherence either kept for 1 day at 21% oxygen level or transferred to 10% oxygen chamber and incubated for 1 day to adapt to lower oxygen. PMH were then treated with 20 mM APAP for 16 h to measure cell death measured by MTT assay or for 3 h to measure reactive oxygen species (ROS) levels by ROS-Glo Assay (Promega), n=1.

SUPPORTING METHODS

Animals

All procedures for animal experiments were approved by the Austrian BMBWF or by the authorities of Baden-Württemberg, Germany. Mice were housed in groups of 2-6 animals in IVC cages in 12:12 h light/dark cycle at central animal facility of the University of Konstanz and had permanent access to water and standard chow ad libitum. C57BL/6 mice (wild type mice, RRID: IMSR_JAX:000664) were originally obtained from Jackson Laboratory and bred in-house. BIM-deficient (*Bim*-/-) mice (B6.129S1-Bcl2l11tm1.1Ast/J, RRID: IMSR_JAX:004525) mice were obtained from Andreas Strasser (Walter and Eliza Hall Institute) and bred in-house. BIM-deficient mice had been backcrossed to the C57BL/6 background over more than 10 generations. NOXA-deficient (*Noxa*-/-) mice (C57BL/6-Pmaip1tm1Ast/J, RRID: IMSR_JAX:011068) were housed as described in Freiburg, Germany. P53-deficient (*p53*-/-) mice (B6.129S2-Trp53tm1Tyj/J, RRID: IMSR_JAX:002101) were housed as described in Innsbruck Austria.

For experiments, 10-12 weeks old mice were fasted for 12 h before i.p. injection of either 500 mg/kg APAP (SIGMA) in warm PBS for 6 h or, 300 mg/kg APAP for 12 h; or an equal volume of warm PBS (SIGMA (control treatment). Injections were always carried out in the morning. As there is a well-established, substantial difference between sexes in the sensitivity to APAP and it is advisable to analyze sexes independently [2], we used for the 6 h experiments only male mice and for the 24 h experiments only female mice. For both setups, additional i.p. injection of 2.5 g/kg glucose (SIGMA) took place 1 h prior to APAP/PBS injection. Blood glucose was measured by punctation of warmed tail vein and direct measurement in a GlucoCheck Excellent glucometer (Activemed). Mice were sacrificed by CO_2 and cervical dislocation, and liver lobes were stored in 10% formalin or shock-frozen in liquid nitrogen for further analysis. Serum from heart blood was obtained by coagulation, and centrifugation for 15 min at 1000 xg. Serum Alanine aminotransferase (ALT) levels were measured using the Reflotron® Plus (Roche) with the respective ALT/GPT test stripes.

Histology and Immunohistochemistry

Fixed and paraffin-embedded murine liver tissue was cut in 4 µm sections, rehydrated and stained by hematoxylin and eosin (H&E) with subsequence dehydration and mounting. For the quantification of necrotic area, three images per samples in 5x magnification were taken blinded and scored blinded using ImageJ area measurement based on a grey value threshold of an 8 bit image measuring percentage of pale, necrotic area while leaving out white blood vessels.

For anti-cleaved caspase-3 or anti-phospho-AMPK immunohistochemistry antigens within rehydrated liver sections were retrieved by boiling in 10 mM Sodium citrate buffer (pH 6) for 15 min. Sections were blocked in 3% hydrogen peroxide/PBS for 60 min, followed by incubation in 5% goat serum, 3% BSA in PBS for additional 60 min. Staining with 1:100 of respective primary antibodies (Cell signaling technology) or goat anti-rabbit-IgG (Jackson Immuno) took place over night at 4 °C, was followed by 3 x 10 min wash in TBS-Tween and incubation with 1:100 biotinylated secondary goat anti-rabbit (Jackson Immuno) for 2 h, followed by additional 3 x 10 min wash and incubation in ABC solution of Vectastain ABC Kit (Biologol). After washing, development was achieved by incubation with 1 x DAB substrate solution (Roche) for 5-10 min and washing in running tab water for 5 min. Counterstaining was performed in hematoxylin for 10 sec, followed by dehydration and mounting. The antibodies used are listed in Supplementary Figure 3.

Isolation of primary murine hepatocytes

To obtain primary mouse hepatocytes (PMH) for *ex vivo* culture, the largest five liver lobes were isolated, short-term stored in 1:75 Heparin/PBS (Sigma-Aldrich) and perfused with 37 °C warm Buffer I (HBSS (Sigma-Aldrich), 0.1% glucose and 2 mM EGTA) for 10 min. To digest the extracellular matrix, additional perfusion with Buffer II (HBSS (Sigma-Aldrich), 5 mM $CaCl_2$ and 0.3 mg/ml Collagenase NB 4 Grade (Nordmark)) for 15-20 min and subsequently incubation for 5 min at 37°C was performed. The capsule of the lobes was disrupted using forceps in DMEM/F-12 Ham (Sigma-Aldrich) media with supplements (10% FCS (Sigma-Aldrich), 4 mM L-glutamine (Sigma-Aldrich), 1 x Pen/Strep 100x (Sigma-Aldrich), 50 µg/ml gentamycin (Sigma-Aldrich), 10

mg/ml Insulin Transferrin Sodium Selenite Supplement (ITS, Roche)) to receive a single cell suspension. After filtering through 100 µM strainer and centrifugation at 75 x g for 5 min, the hepatocyte pellet was resuspended in 40% Percoll (GE Healthcare)/medium and centrifuged at 500 x g for 10 min to remove dead cells. Live cells in the pellet were resuspended, counted, and seeded per well with 0.015 x 10^6 cells per well for 96-well and Seahorse plate, 0.1 x 10^6 cells/12-well, 0.4 x 10^6 cells/6-well, 1.5 x 10^6 cells/10-cm dish and 0.045 x 10^6 cells/8-well IBIDI glass slide. All plates, dishes and slides were pre-coated with 0.3 mg/ml collagen (Sigma-Aldrich) for minimally 2 h prior to seeding. Hepatocytes were kept in culture at 37 °C with 5% CO_2 with daily medium exchange and not longer than 3 days.

Isolation of primary human hepatocytes

Isolation of human hepatocytes was performed at the Insel University Hospital, as described previously[3]. Normal human liver tissue was taken from the periphery of liver specimens from patients undergoing surgical resection for colorectal metastases. Informed consent of the patients was obtained in accordance with institutional guidelines and the local ethics committee. Briefly, cells were isolated using a two-step enzymatic perfusion protocol. The viability of the isolated hepatocytes was determined by trypan blue exclusion, and only preparations of over 90% viability were used. The hepatocytes were seeded onto rat tail collagen-coated tissue culture plastics in DMEM containing 10% FCS, left to attach for 1 to 2 h, and then washed twice with phosphate-buffered saline (PBS) to remove unattached cells. The hepatocytes were cultured in arginine-free Williams E medium (Gibco) supplemented with insulin (0.015 IU/mL), hydrocortisone (5 µmol/L), penicillin (100 IU/mL), streptomycin (100 µg/mL), glutamine (2 mmol/L), and ornithine (0.4 mmol/L) for 24 h before use.

Cell lines and culture

Immortalized human hepatocytes (IHH, RRID: CVCL_8278) cells were cultured in same DMEM/F12 medium as PMH. Hepatoma HepG2 (RRID: CVCL_0027) cells were cultured in DMEM medium (Sigma-Aldrich, 10% FCS, 2 mM L-glutamine, 20 µg/ml gentamycin) at 37 °C with 5% CO_2. After detachment by Trypsin/EDTA (Sigma-Aldrich), cells were counted and seeded per

well with 0.015 x 10^6 cells/96-well and Seahorse plate, 0.4 x 10^6 cells/6-well, 1.5 x 10^6 cells/10-cm dish and 0.035 x 10^6 cells/8-well IBIDI glass slide for IHH, and 0.03 x 10^6 cells/96-well and Seahorse plate, 0.6 x 10^6 cells/6-well, 2 x 10^6 cells/10-cm dish and 0.045 x 10^6 cells/8-well IBIDI glass slide for HepG2 cells. For experiments with no or low glucose conditions, SILAC Advanced DMEM/F-12 Flex Media (Gibco) supplemented with 2% FCS (Sigma-Aldrich), 4 mM L-glutamine (Sigma-Aldrich), 1 x Pen/Strep (Sigma-Aldrich), 50 µg/ml gentamycin (Sigma-Aldrich), 15 mM HEPES, 0.847 mM L-Arginine (Sigma-Aldrich) and 0.624 mM L-Lysine (Sigma-Aldrich) was used and optionally adjusted to desired glucose concentration.

CRISPR/Cas9-mediated deletion was achieved by transduction of cell lines with lentiviruses containing respective guide-RNAs previously produced by transfection of HEK293T cells (RRID: CVCL_0063). Guide-RNA oligonucleotides were cloned into lentiCRISPRv2 backbone as described before[4], amplified in bacteria and confirmed by sequencing at Eurofins Genomics. The resulting plasmid together with pCMVdR8.2 and pMD2.VSV-G, was transfected in HEK293T cells with ROTI®Fect PLUS (Roth) according to manufacturer's protocol. Virus-containing supernatant was collected 1-2 days post transfection and administered in combination with 12 µg/ml Polybrene (Hexadimethrine bromide, Sigma-Aldrich) to IHH and HepG2 target cells for 1 day. Selection with 1 µg/ml puromycin took place for 1-2 weeks. The used guide RNA oligonucleotides are listed in Supplementary Table 1.

GEV16-mediated inducible overexpression of BCL-2, BCL-x_L or MCL-1 was achieved as described in Vince et al.[5]. In brief, two-step cell line generation was performed by first transduction of target cells with HEK293T-dervided virus containing the GEV16 plasmid (pHCA/GAL4(1-93).ER.VP16) and generation of a stable cell line by selection with hygromycin B. The second step included virus production and transduction of generated GEV16-cells with expression plasmids (pF-5xUAS-MCS-SV40-Puro-based) containing either human BCL-2, BCL-x_L and MCL-1 genes, each tagged with StrepIII. Cells were further selected with 1 µg/ml puromycin. Overexpression of target genes was achieved by treatment of cells with 60 nM 4-hydroxytamoxifen (4-HT) 24 h prior to experiments.

Cell death Assay (MTT)

Cell death of primary murine and human hepatocytes was measured by 3-(4,5-dimethylthiazol-2-yl)-2,5-diphenyltetrazolium bromide (MTT, Sigma-Aldrich) viability assay. After 1 h incubation in 10% MTT/media solution, violet formazan crystals were dissolved in DMSO, and absorbance was measured at 562 nm with the Infinite® 200 PRO plate reader (TECAN). Data was normalized to untreated control.

PI-Hoechst staining

After treatment, cells were stained with 5 µg/ml propidium iodide (PI) (Sigma-Aldrich) and 5 µg/ml Hoechst33342 (BioRad) for 30 min at 37 °C. Fluorescence was measured with the Infinite® 200 PRO plate reader (TECAN) from the top with automatically determined Z-position, in circular 4x4 measurements per well and, optimized gain for red (ex: 535 nm, em: 617 nm) and blue channel (ex: 361 nm, em: 486 nm). The ratio of PI and Hoechst fluorescence per measurement was determined and normalized to untreated control.

Annexin V-PI staining

Cell death in IHH and HepG2 cells was assessed by Annexin V-FITC and PI staining, and flow cytometry analysis. Cells were collected in a V-bottom 96-well plate and pelleted at 500 x g for 10 min with subsequent staining with 100 µl/well self-made Annexin V-FITC (1:1000) in binding buffer (10 mM HEPES, 150 mM NaCl, 5 mM KCl, 1 mM $MgCl_2$, 1.8 mM $CaCl_2$) for 20 min at 4 °C. After centrifugation, cell pellets were resuspended 100 µl/well binding buffer containing 1 µg/ml PI and directly measured in the BD LSR Fortessa™ flow cytometer. Analysis was performed using FlowJo software by gating for single cells being positive for FITC or PI.

Quantitative RT-PCR

RNA isolation was archived by lysing samples in peqGOLD TriFast™ (VWR) or TRIzol™ Reagent (Invitrogen) according to manufacturer’s protocol. Small pieces of frozen murine liver were lysed in 1 ml by 4 Hz shaking in the TissueLyzer II (QIAGEN). Harvested cell pellets were directly lysed in 1 ml peqGOLD TriFast™ (VWR) or 400 µl TRIzol™ Reagent (Invitrogen). After isolation of RNA,

chromosomal DNA was digested with DNase I t (NEB), and 2 µg of DNA-free RNA were reverse transcribed into cDNA by High Capacity cDNA Reverse Transcription Kit (Applied Biosystems). Real-Time-quantitative PCR was performed on a StepOnePlus™ qPCR device (Applied Biosystems) using SYBR® Green (Applied Biosystems) and specific primers. Murine or human beta-actin C_T values were used for normalization of respective samples (ΔC_T(gene) = C_T(gene) – C_T(beta-actin)). The used primers are listed in Supplementary Table 2.

Western Blot analysis

For Western Blot, small pieces of murine liver were homogenized in 1 ml RIPA Buffer (50 mM Tris pH 7.4, 150 mM NaCl, 0.1% SDS, 1% NP-40, 0.5% sodium deoxycholate, fresh 1x cOmplete™ Protease Inhibitor Cocktail (Roche)) by 4 Hz shaking in the TissueLyzer II (QIAGEN). Cultured cells were harvested from 6-well plates using Trypsin/EDTA (Sigma-Aldrich) and lysed in 100-200 µl RIPA Buffer on ice for 20 min. Lysates were centrifuged for 20 min at 14.000 x g, 4 °C and total protein concentration of supernatant was determined using Pierce™ BCA Assay Kit (Thermo Fisher). Samples for Western Blotting were boiled in 1 x Laemmli buffer (5x: 5% SDS, 0.2 mM EDTA, 0.02% Bromophenol blue, 125 mM Tris pH 6.8, 50% Glycerol, 160 mM DTT) for 5 min at 95 °C and equal protein amounts (30-50 µg/sample) were loaded into 8-15% SDS-acrylamide gels (stacking gel: 5% Rotiphorese® 30 Gel (Roth), 0.13 M Tris-HCl pH 6.8, 0.1% SDS, 0.1% APS, 0.01% TEMED; separation gel: 12% Rotiphorese® 30 Gel (Roth), 0.38 M Tris-HCl pH 8.8, 0.01% SDS, 0.01% APS, 0.08% TEMED). After SDS-Polyacrylamide-gel electrophoresis (SDS-PAGE), proteins were wet-blotted on a PVDF membrane (Roche) in Blotting Buffer (25 mM Tris, 192 mM glycine, 0.1% SDS, 21.5% methanol), unspecific binding was blocked in 5% non-fat dry milk (Rapilait MIGROS)/TBS-T (137 mM NaCl, 2.7 mM KCl, 15 mM Tris, 0.1% Tween-20) for 1 h and the membrane was incubated with primary antibodies in 5% BSA/TBS-T over night at 4 °C. After 3 x 5 min wash intervals in TBS-T, incubation with peroxidase-conjugated secondary antibodies in 5% BSA/TBS-T took place for 2 h at room temperature with additional washing afterwards. For visualization, ECL solution (250 mM Luminol, 90 mM p-coumaric acid, 10 mM Tris) with 1% hydrogen peroxide was applied on membranes and

proteins were detected in Image Quant LAS4000 (GE Heatlhcare Life Sciences). The used primary and secondary antibodies are listed in Supplementary Table 3.

Immunoprecipitation

For immunoprecipitation (IP), cells were harvested from 10 cm dishes using Trypsin/EDTA, washed with PBS, lysed in 400 µl CHAPS buffer (50 mM Tris, 150 mM NaCl, 0,5% deoxy-cholic acid, 1% CHAPS, 1x cOmplete™ Protease Inhibitor Cocktail (Roche), pH 8.0) on ice for 30 min with subsequent centrifugation and protein determination as described in Western Blot method. As input control, 10% of supernatant was directly boiled in 1 x Laemmli buffer as described. Remaining supernatant was incubated with bait or isotype-antibodies for 2-3 hours at room temperature under constant rotation. Per sample 30 µl Protein G Sepharose 4 Fast Flow beads (GE Healthcare Life Sciences) were 3x times in CHAPS buffer by centrifugation at 14 000 x g for 1 min each. Beads were added to samples and incubated overnight at 4 °C under constant rotation. On next day, beads were washed 3x to remove unbound proteins and boiled in 2x Laemmli buffer at 95°C for 10 min to release bound proteins. Equal volumes were used for Western Blotting.

Cytosolic and mitochondrial fractionation

To study mitochondrial outer membrane permeabilization, mitochondrial and cytosolic fractions were collected by harvesting cells from 10-cm dishes using Trypsin/EDTA, lysing in 200 µl cold cytosolic extraction buffer (1.4 mM KH_2PO_4 ph7.2, 4.3 mM Na_2HPO_4, 250 mM sucrose, 70 mM KCl, 137 mM NaCl, 200 µg/ml digitonin, 1x cOmplete™ Protease Inhibitor Cocktail (Roche)) on ice for 5 min and collecting the supernatant (cytosolic fraction) after centrifugation for 10 min at 1000 x g, 4 °C. The pellet containing mitochondria was washed once with cytosolic extraction buffer by additional centrifugation and thereafter lysed in 200 µl mitochondrial lysis buffer (50 mM Tris pH 7.4, 150 mM NaCl, 2 mM EDTA, 2 mM EGTA, 0,2% Triton-X-100, 0,3% NP-40, 0,5% deoxycholic acid, 1x cOmplete™ Protease Inhibitor Cocktail (Roche)) on ice for 10 min. Mitochondrial faction was collected by centrifugation for 10 min at 14000 x g, 4 °C. Samples were

boiled in 1 x Laemmli buffer as described before and used in a 4:1 ratio (cytosolic/mitochondria) for Western Blotting.

FADU Assay

Early DNA damage was assessed by automated Fluorimetric Detection of Alkaline DNA Unwinding (FADU) Assay as described by Moreno-Villanueva [6]. In short, cells were seeded and treated in specially prepared 96-well plates and placed in a pipetting robot that conducted cell lysis, alkaline-mediated unwinding of DNA, neutralization step and SYBR Green® administration. Amount of double-stranded DNA was determined in fluorescent plate reader (ex: 485 nm, em: 530 nm) with subsequent calculation of P (sample conditions) to T ratios (100% dsDNA condition) and normalization to untreated control.

GSH measurement

To determine intracellular glutathione (GSH) levels, serving as indicator for oxidative stress, cells were scratched from 96-well plate in 100 µl/well of 1% sulfosalicylic acid in water and 20 µl of the properly mixed lysate were transferred to new 96-well plate, diluted with 80 µl/well deionized water, and mixed with 100 µl/well GSH Reaction Buffer (GSH buffer (100 mM Na_2HPO_4, 100 µM EDTA), 100 µM DTNB, 200 µM NADPH and 5 µl of 500 U glutathione reductase(Sigma-Aldrich)). Absorption at 405 nm was measured directly every 5 min for 1 h at 37 °C in the Infinite® 200 PRO plate reader (TECAN). Slope of increasing absorbance was normalized to untreated controls.

Seahorse ATP Rates and Mito Stress Test

To quantify the cellular bioenergetic profile comprising glycolytic and mitochondrial ATP generation, Seahorse XF Real-Time (Induced) ATP Rate Assay Kit (Agilent) was used according to manufacturer’s protocol. For the Induced ATP Rate Assay, following adaptions were implemented: In brief, cells were seeded one day prior to experiments into Seahorse XFe24 cell culture plates, medium was carefully replaced to Seahorse XF DMEM medium pH 7.4 (Agilent) supplemented with 18 or 36 mM glucose, 1 mM pyruvate and 2 mM L-glutamine, and cells were incubated at 37°C without CO_2 for 60 min. Directly before measurement, medium was changed

again. Seahorse extracellular Flux Analyzer XFe24 (Agilent) was started with pre-hydrated XFe24 sensor cartridge containing, APAP solved in Seahorse XF DMEM Medium pH 7.4 in Port A and B (final concentration together: 15 mM), oligomycin in Port C (final 1.5 µM), a mix of rotenone, antimycin A (both final concentration 0.5 µM) and Hoechst33342 (final 1 µg/ml) in Port D. After Injection of Port A and B, cells were incubated and measured for 1 h before the program continued with oligomycin injection. For the Non-Induced Assay, both APAP ports were excluded, and others moved forward. Afterwards, the cell number was determined based on Hoechst33342-positive nuclei using an automated algorithm in Cellomics ArrayScan™ VTI High-Content Screening (Cellomics™). Nuclei were identified based on shape, intensity, and size. Data analysis was performed with Agilent Seahorse Analytics Online Software by normalizing to cell number and to 100% ATP production of untreated wild type control, cultured in normal glucose medium. For calculation of 'maximal ECAR' and basal and maximal respiration in IHH cells, the Seahorse XF Cell Mito Stress Test Kit (Agilent) was used according to manufacturer's protocol with normalization to cell count as described above. The 'maximal ECAR' was calculated as mean of ECAR after oligomycin injection, thus after inhibiting mitochondrial ATP production. Likely, the near-constant ECAR in no glucose is attributed to non-glycolytic acidification, while the increase in ECAR in the normal glucose condition is due to increased glycolysis.

ATP measurement (CellTiter-Glo®)

Intracellular ATP levels were determined with the CellTiter-Glo® 2.0 Assay (Promega) as described in the manufacturers' instructions. Briefly, cells in 96-well plate were lysed with CellTiter-Glo® Reagent and ATP-dependent luminescence was measured after 10 min in Infinite® 200 PRO plate reader (TECAN).

Lactate measurement

Lactate secretion was measured using the LAC 142 Kit (Diaglobal) and the Diaglobal Photometer the according to manufacturers' protocol. For sampling, medium of cells seeded in 6-well plates was changed and supernatant samples were taken at 0, 2, 6 and 24 h. Lactate secretion per sample was calculated as increase normalized to untreated control.

Mitochondrial Morphology by MitoTracker™ confocal microscopy

MitoTracker™ Green (Invitrogen) fluorescent dye was used to monitor mitochondrial morphology. Cells seeded in 8-well glass bottom IBIDI chambers slides were stained with 100 nM MitoTracker™ Green in phenol red-free DMEM/F-12 medium with supplements for 30 min at 37°C, followed by three times washing with warm medium and further incubation for 30 min at 37°C. Live cell imaging at 37°C, 5% CO_2 was performed with the LSM 880 AxioObserver (Zeiss) at the Bioimaging Center (University of Konstanz) with 488 nm Laser (ex: 488 nm, em: 515 nm) and Plan-Apochromat 40x/1.4 Oil DIC M27 objective. Images were acquired with the Zen Black software (Zeiss) and subsequently processed equally with Zen Blue. Mitochondrial morphology was quantified with ImageJ software by calculating the ratio length/width from 30 randomly selected mitochondria per picture (4-9 images per sample, n=3), grouping them into hyperfused (ratio>10), fused (ratio≥5<10) and fragmented (ratio<5) morphology and, finally calculating the percentages per sample.

Mitochondrial ROS by MitoSOX™ confocal microscopy

Mitochondrial reactive oxygen species can be visualized via MitoSOX™ Red (Invitrogen), a fluorogenic dye, which is specifically oxidized by mitochondrial superoxide in living cells. Cells seeded in 8-well glass bottom IBIDI chambers slides were stained with 5 µM MitoSOX™ Red in phenol red-free DMEM/F-12 medium with supplements for 15 min at 37°C and 5% CO_2, followed by washing with warm medium, and live cell imaging at 37°C, 5% CO_2 was performed immediately with the LSM 880 AxioObserver (Zeiss) at the Bioimaging Center (University of Konstanz) with 514 nm Laser (ex: 514 nm, em: 593 nm) and Plan-Apochromat 40x/1.4 Oil DIC M27 objective. Images were acquired with the Zen Black software (Zeiss) and subsequently processed equally with Zen Blue. The mean fluorescence intensity of each cell in a picture was quantified using the ImageJ software and mean of MFI per technical sample was determined.

MitoSOX™ measurement by flow cytometry

Quantification of mitochondrial ROS in HepG2 and splenocytes was assessed by MitoSOX™ Red (Invitrogen) staining and flow cytometry analysis. Optionally treated cells were harvested using

Trypsin/EDTA (Sigma-Aldrich), distributed in V-bottom 96-well plate, and stained with 5 µM MitoSOX^{TM} Red for 10 min at 37 °C. After washing in FACS Buffer (2 mM EDTA, 5% BSA, PBS), cells were resuspended and measured in FACS Buffer containing 1 µg/ml DAPI on a LSR Fortessa™ flow cytometer (BD Biosciences). HepG2 cells were measured in High Throughput Sampler (HTS). Analysis of the Red Median Fluorescence Intensity of single, live cells was done using FlowJo software.

MitoSOX^{TM} measurement with Incucyte®

To study the time course of mitochondrial ROS production, cells were pre-conditioned with glucose for 1.5 h, then stained with MitoSox^{TM} (Invitrogen) in phenol red-free medium with respective glucose concentrations for 15 min, followed by APAP treatment to obtain final concentrations of 5 µM MitoSox^{TM}. Cells were incubated 24 h at 37°C 5% CO_2 in the Incucyte® SX1 system and in intervals of 1 h images with 10x magnification were taken. Images were analyzed using Incucyte® 2021B Software by solely quantifying cytosolic signals (nuclear staining was excluded) and normalizing the measured integrated intensity to the cellular confluence.

Mitochondrial Potential by TMRE staining

For quantification of mitochondrial membrane polarization, cells were harvested by Trypsin/EDTA treatment (Sigma-Aldrich) and stained with 100 nM Mitotracker^{TM} Green and 20 nM TMRE (Sigma-Aldrich) in FACS Buffer (2 mM EDTA, 5% BSA, PBS) for 30 min at 37 °C. After adding DAPI (final concentration 1 µg/ml), samples were directly measured on a LSR Fortessa™ flow cytometer. HepG2 cells were measured in High Throughput Sampler (HTS). Percentage of the single, live, MitoTracker^{TM} Green-positive, and TMRE-positive cells were determined using FlowJo software.

Acridine Orange staining and quantification

To visualize acidic lysosomes, cells were seeded in 8-well glass bottom IBIDI chambers slides, treated, and then stained with 3 µM acridine orange (Sigma-Aldrich) and 5 µg/ml Hoechst33342 for 30 min at 37 °C. Afterwards, cells were carefully washed twice with phenol red-free DMEM/F12

and visualized on an AxioObserver microscope using AxioVision software (Zeiss). Acridine Orange stains acidic components and when applied ad high concentration it can be used to distinguish between green-fluorescent DNA (monomer molecules) and red-fluorescent lysosomes (stacked molecule). Quantification of lysosomes was achieved by taking four images per samples in 20x magnification and blindly determining bright red area using ImageJ area measurement based on a grey value threshold of 8-bit images. To avoid cell density bias, red area (lysosomes) was normalized to blue area (nuclei) per sample. Ratio was normalized to untreated, normal glucose control.

Lysotracker and Cathepsin B staining

To visualize functional lysosomes, cells were seeded in 8-well glass bottom IBIDI chambers slides, treated and then stained with 50 nM LysoTracker^{TM} Green DND-26 (Invitrogen), 1:26 dilution of Cathepsin B Magic Red^{TM} (BIO-RAD), and 5 µg/ml Hoechst33342 (BIO-RAD) for 60 min at 37°C. Before visualization on a AxioObserver microscope, cells were carefully washed twice with phenol red-free DMEM/F12 media.

Single cell RNA sequencing analysis

Annotated single cell RNA sequencing data of APAP-treated mice was downloaded from Kolodziejczyk et al.[1] and analyzed in R environment using R Studio. After initial filtering of the expression matrix as described in Kolodziejczk et al., the R package Seurat (version 4.1.0) was used to normalize, find variable genes, scaling and dimensional reduction (PCA) as described in Butler et al. Clustering was not performed but the provided annotated cell clusters were re-applied and re-checked based on cluster-specific expression patterns. Single cell datasets were visualized using Uniform Manifold Approximation and Projection (UMAP) dimensional reduction using RunUMAP and DimPlot function. Specifically, hepatocyte subset of SPF-housed mice treated with PBS or APAP were analyzed and compared (described in Kolodziejczyk et al., 2020)[1]. Differentially expressed genes (DEGs) between hepatocytes clusters were calculated using FindMarker function with p-value <0.05. Gene set enrichment and gene ontology/pathway

analysis were performed with hepatocyte DEGs using the R packages clusterProfiler (version 4.0.5) and enrichplot (version 1.12.3).

Bulk hepatocyte RNA sequencing analysis

Age and sex-matched C57BL/6J wild type mice were fasted overnight with unlimited access to water. Control animals were not fasted (unfasted) and had permanent access to food and water ad libitum. Following the fasting period, mice were sacrificed, the liver lobes were collected, and total RNA was isolated using the SV Total RNA Isolation System (Promega). Paired-end bulk mRNA sequencing (mRNA-Seq) was performed by the Next Generation Sequencing (NGS) Platform of the University of Bern, Switzerland. Obtained raw paired-end data was processed using the public server usegalaxy.org with Trimmomatic, STAR, and featureCounts tools for the generation of gene counts. Quality control was repeatedly checked using FastQC and MultiQC tools. The generated count matrix was downloaded and further analyzed in R environment using R studio. Gene counts were filtered, normalized, log-transformed and DEGs were calculated with an adjusted p-value <0.05 using the R package DESeq2 (version 1.32.0). Visualization was performed using the R package "EnhancedVolcano" (version 1.10.0) and pheatmap (version 1.0.12). Gene set enrichment, gene ontology/pathway and cluster net analysis were performed using the R packages clusterProfiler (version 4.0.5) and enrichplot (version 1.12.3).

Statistics and data display

If not stated otherwise, data obtained from *in vivo* experiments are median ± standard deviation (SD) and data from *ex vivo* and *in vitro* experiments are means ± SD of a minimum of three independent experiments with several technical replicates. GraphPad Prism 8 software was used to perform statistical analysis and data visualization. Statistical significance was calculated by unpaired two-tailed Student's test when comparing two groups, One-Way or Two-Way ANOVA when comparing three or more dependent groups, respectively. Suitable post-hoc tests (Tukey's or Sidak's) were applied and are stated in respective figure legends. Statistical significance is displayed as p-values with ≤ 0.05 being statistically significant (* = $p < 0.05$, ** = $p < 0.01$, *** = $p < 0.001$, **** = $p < 0.0001$).

All RNA sequencing dataset analyses were performed in the R environment using R studio (version 4.1.1).

Data and code availability

All raw and processed data as well as bioinformatic codes that were used to generate results reported in the manuscript are available upon reasonable request.

SUPPORTING MATERIAL

Supplementary Table 1: Mice.

NAME	SOURCE	IDENTIFIER/STRAIN	SEX	AGE
C57BL/6J = Wildtype, WT	Jackson Laboratory	RRID: IMSR_JAX:000664	M+F	10-12 weeks
C57BL/6 Noxa-/- (C57BL/6-Pmaip1tm1Ast/J)	[7];	RRID: IMSR_JAX:011068	M+F	10-12 weeks
C57BL/6 Bim-/- (B6.129S1-Bcl2l11tm1.1Ast/J)	[8]	RRID: IMSR_JAX:004525	M+F	10-12 weeks
C57BL/6 p53-/- (B6.129S2-Trp53tm1Tyj/J)	[9];	RRID: IMSR_JAX:002101	M+F	10-12 weeks

Supplementary Table 2: Cell lines.

NAME	SOURCE	IDENTIFIER
IHH (Immortalized Human Hepatocytes)	Gift from J.F. Dufour, Bern	RRID: CVCL_8278
HepG2 (Hepatocellular carcinoma cells, human)	Gift from J.F. Dufour, Bern	RRID: CVCL_0027
HEK293T (Human Embryonic Kidney cells)	Gift from M. Gröttrup, Konstanz	RRID: CVCL_0063

Supplementary Table 3: Oligonucleotides for guide RNA for CRISPR/Cas9 Knockout Generation.

REAGENT or RESOURCE	SOURCE
TP53 (p53) exon 2<br>Fw: CACCGTCGACGCTAGGATCTGACTG;<br>rev: AAACCAGTCAGATCCTAGCGTCGAC	This paper
MAPK11 (p38) exon2<br>Fw: CACCGCACAAAAACGGGGTTACGTG;<br>rev: AAACCACGTAACCCCGTTTTTGTGC	This paper
BCL2L11 (BIM)<br>Fw: CACCGAATCCTGAAGGCAATCACGG;<br>rev: AAACCCGTGATTGCCTTCAGGATTC	This paper
DNM1L (DRP1) exon 12<br>Fw: CACCGACAAGTCACCACTTCAACTA;<br>rev: AAACTAGTTGAAGTGGTGACTTGTC	This paper
MFN1 exon 2<br>Fw: CACCGGTGGCTATTCGATCAAGTTC;<br>rev: AAACGAACTTGATCGAATAGCCACC	This paper
MFN2 exon 4<br>Fw: CACCGGAACAGGTTCTGGACGTCAA;<br>rev: AAACTTGACGTCCAGAACCTGTTCC	This paper
BAX<br>CAAGCGCATCGGGGACGAAC	[10]
Bak<br>ACGGCAGCTCGCCATCATCG	[10]

Supplementary Table 4: Oligonucleotides for forward (fw) and reverse (rev) primers for RT-qPCR.

REAGENT or RESOURCE	SOURCE
mBcl2l11 (BIM)<br>Fw: GCCAGGCCTTCAACCACTAT;<br>rev: TGCAAACACCCTCCTTGTGT	This paper

mPmaip1 (NOXA)<br>Fw: ATAACTGTGGTTCTGGCGCA;<br>rev: TCCTTCAAGTCTGCTGGCAC	This paper
mBbc3 (PUMA)<br>Fw: AGGTGCCTCAATAGCAACCC;<br>rev: CTCCCTGGAGCCCCG	This paper
hBCL2L11<br>Fw: TAAGTTCTGAGTGTGACCGAGA;<br>rev: GCTCTGTCTGTAGGGAGGTAGG	This paper
hPMAIP1<br>Fw: GTGCCCTTGGAAACGGAAGA;<br>rev: CCAGCCGCCCAGTCTAATCA	This paper
hBBC3<br>Fw: GAGCGGCGGAGACAAGAG;<br>rev: TAAGGGCAGGAGTCCCATGA	This paper
mCdkn1a (p21)<br>Fw: CAGCAGATTAAAAGGTGCCACAG;<br>rev: AGAGTGCAAGACAGCGACAA	This paper
mMdm2<br>Fw: GGCCTCCAGGTTAGACCAAA;<br>rev: ACTCGGGACTCCAAACACATC	This paper
mGadd45b<br>Fw: GATGAATGTGGACCCCGACA;<br>rev: TCCCAGAAGGTATCACGGGT	This paper
mBax<br>Fw: GCACGTCCACGATCAGTCA;<br>rev: CGATCCTGGATGAAACCCTGT	This paper
mDnm1l (DRP1)<br>Fw: GGGCACTTAAATTGGGCTCC; rev:<br>TGTATTCTGTTGGCGTGGAAC	[11]
mFis1<br>Fw: GGCTGTCTCCAAGTCCAAATC;<br>rev: GGAGAAAAGGGAAGGCGATG	[11]
mOpa1<br>Fw: TCACCTCTGCGTTTATTTGAAGA;<br>rev: GGGTAGAACGGGAGGAAAGG	[11]
mMfn1<br>Fw: TATCGATGCCTTGCGGAGAT;<br>rev: GGCGAATCACAACACTTCCA	[11]
mMfn2<br>Fw: GGAGACCAACAAGGACTGGA;<br>rev: TGCACAGTGACTTTCAACCG	[11]
mCox2 (mitochondrial DNA)<br>Fw: CAGTCCCCTCCCTAGGACTT;<br>rev: TCAGAGCATTGGCCATAGAA	This paper
mAtf6 (mitochondrial DNA)<br>Fw: CACTGGCACCTTCACCAAAAT;<br>rev: AGGCGTTTTGAGGATGGGAA	This paper
mCyp2e1<br>Fw: TGGACGCTGTAGTGCATGAG;<br>rev: AACTGTACCCTTGGGGATGAC	This paper
mGpx1<br>Fw: ATCAGTTCGGACACCAGAATGG;<br>rev: TGATGTACTTGGGGTCGGTC	This paper
mHo1<br>Fw: CCAGAGAAGGCTTTAAGCTGGT;<br>rev: CCTCGTGGAGACGCTTTACA	This paper
mNqo1<br>Fw: CCATGTACGACAACGGTCCT;<br>rev: ACGCAGGATGCCACTCTGAA	This paper

mBnip3<br>Fw: TTCTCACTGTGACAGCCCAC;<br>rev: TCTTCCTCAGACAGAGTGCT	This paper
mUlk1<br>Fw: CAGCAGACAGCCAGGTCC;<br>rev: CCACTTGGGGAGAAGGTGTG	This paper
mPink1<br>Fw: CACACTGTTCCTCGTTATGAAGA;<br>rev: CTTGAGATCCCGATGGGCAAT	[12]
mPrkn<br>Fw: TTGCTGGGACGATGTCTTAATTC;<br>rev: AAAGCTACCGACGTGTCCTTG	[12]
mBeclin<br>Fw: TCAGCCGGAGACTCAAGGT;<br>rev: CACAGCGGGTGATCCACATC	[13]
mPgc1a<br>Fw: AAACTTGCTAGCGGTTCTCA;<br>rev: TGGCTGGTGCCAGTAAGAG	[13]
mTfb2m<br>Fw: TCCACATTTGGAGCCCTTAC;<br>rev: GAACACCTGCTGACCAAGGA	This paper
mActb<br>Fw: GATCAAGATCATTGCTCCTCCTG;<br>rev: CAGCTCAGTAACAGTCCGCC	This paper
hACTB<br>Fw: CATGTACGTTGCTATCCAGGC;<br>rev: CTCCTTAATGTCACGCACGAT	This paper

Supplementary Table 5: Primary and secondary antibodies for Western Blot and Immunoprecipitation.

REAGENT or RESOURCE	SOURCE	IDENTIFIER
Mouse monoclonal anti-alpha-Tubulin (B-5-1-2)	Sigma-Aldrich	Cat: #T5168;<br>RRID: AB_477579
Mouse monoclonal anti-Caspase 9 (5B4)	Enzo Life Sciences	Cat: #ADI-AAM-139;<br>RRID: AB_10614959
Mouse monoclonal anti-Cytochrome c (7H8.2C12)	BD Pharmigen™	Cat: #556433;<br>RRID: AB_396417
Mouse monoclonal anti-Mcl-1 (G-7)	Santa Cruz Biotechnology	Cat: # sc-74437;<br>RRID: AB_1126070
Mouse monoclonal anti-Noxa (114C307.1)	Enzo Life Sciences	Cat: # ALX-804-408-C100;<br>RRID: AB_2052079
Mouse monoclonal anti-p21 (F-5)	Santa Cruz Biotechnology	Cat: #sc-6246;<br>RRID: AB_628073
Mouse monoclonal anti-Parkin antibody (PRK8)	Santa Cruz Biotechnology	Cat# sc-32282; RRID: AB_628104
Rabbit monoclonal anti-Bak (D4E4)	Cell Signaling Technology	Cat: #12105;<br>RRID: AB_2716685
Rabbit monoclonal anti-Bcl-2 (50E3)	Cell Signaling Technology	Cat: #2870;<br>RRID: AB_2290370
Rabbit monoclonal anti-Bcl-xl (54H6)	Cell Signaling Technology	Cat: #2764;<br>RRID: AB_2228008
Rabbit monoclonal anti-Bim (C34C5)	Cell Signaling Technology	Cat: #2933;<br>RRID: AB_1030947
Rabbit monoclonal anti-DRP1 (D6C7)	Cell Signaling Technology	Cat: #8570;<br>RRID: AB_10950498
Rabbit monoclonal anti-Hexokinase-1 (C35C4)	Cell Signaling Technology	Cat: #2024;<br>RRID: AB_2116996

Rabbit monoclonal anti-Hexokinase-2 (C64G5)	Cell Signaling Technology	Cat: #2867; RRID: AB_2232946
Rabbit monoclonal anti-Mitofusin-1 (D6E2S)	Cell Signaling Technology	Cat: #14739; RRID: AB_2744531
Rabbit monoclonal anti-Mitofusin-2 (D2D10)	Cell Signaling Technology	Cat: #9482; RRID: AB_2716838
Rabbit monoclonal anti-p53 (7F5)	Cell Signaling Technology	Cat: #2527; RRID: AB_10695803
Rabbit monoclonal anti-PARP (46D11)	Cell Signaling Technology	Cat: #9532; RRID: AB_659884
Rabbit monoclonal anti-phospho-4EBP1 (Thr37/46) (236B4)	Cell Signaling Technology	Cat: #2855; RRID: AB_560835
Rabbit monoclonal anti-phospho-AMPK (Thr172) (40H9)	Cell Signaling Technology	Cat: #2535; RRID: AB_331250
Rabbit monoclonal anti-phospho-JNK (Thr183/Tyr185) (81E11)	Cell Signaling Technology	Cat: #4668; RRID: AB_823588
Rabbit monoclonal anti-phospho-mTOR (Ser2448) (D9C2)	Cell Signaling Technology	Cat: #5536; RRID: AB_10691552
Rabbit monoclonal anti-phospho-p38 (Thr180/Tyr182) (D3F9) XP	Cell Signaling Technology	Cat: #4511; RRID: AB_2139682
Rabbit polyclonal anti-Bax	Cell Signaling Technology	Cat: #2772; RRID: AB_10695870
Rabbit polyclonal anti-Caspase 3	Cell Signaling Technology	Cat: #9662; RRID: AB_331439
Rabbit polyclonal anti-cleaved Caspase 3 (Asp175)	Cell Signaling Technology	Cat: #9661; RRID: AB_2341188
Rabbit polyclonal anti-cleaved Lamin A	Cell Signaling Technology	Cat: #2035; RRID: AB_2234647
Rabbit polyclonal anti-LC3B	Sigma-Aldrich	Cat: # L8918; RRID: AB_1079382
Rabbit polyclonal anti-VDAC	Cell Signaling Technology	Cat: #4866; RRID: AB_2272627
Peroxidase AffiniPure Goat Anti-Mouse IgG	Jackson Immuno Research	Cat: #115-035-174; RRID: AB_2338512
Peroxidase AffiniPure Goat Anti-Rabbit IgG	Jackson Immuno Research	Cat: #111-035-144; RRID: AB_2307391
Mouse IgG1 kappa Isotype Control (P3.6.2.8.1)	Invitrogen, eBioscience ™	Cat: #14-4714-82; RRID: AB_470111
ChromPure Rabbit IgG, whole molecule	Jackson Immuno Research	Cat: # 011-000-003; RRID: AB_2337118

Supplementary Table 6: Plasmids.

NAME	SOURCE	IDENTIFIER
LentiCRISPRv2 (CRISPR/Cas9 plasmid)	[4,14]	Addgene Plasmid Cat: #52961
pCMV-VSV-G (packaging plasmid for CRISPR/Cas9)	Gift from Marco Herold, Melbourne	Addgene Plasmid Cat: #8454
psPAX2 (envelope plasmid for CRISPR/Cas9)	Gift from Marco Herold, Melbourne	Addgene Plasmid Cat: #12260
pHCA/GAL4(1-93).ER.VP16 (GEV16 plasmid)	[15]; Gift from J. Silke, Melbourne	Addgene Plasmid Cat: #108216
pF-5xUAS-MCS-SV40-Puro (expression backbone with multiple cloning site)	Gift from J. Silke, Melbourne	Addgene Plasmid Cat: #165891
pF-MCS-HA-StrepIII-MCL1 (StrepIII-tagged Mcl-1 overexpression plasmid, cloned from from pF-5xUAS-MCS)	This paper	N/A
pF-MCS-HA-StrepIII-Bclxl (StrepIII-tagged Bcl-xl overexpression plasmid, cloned from from pF-5xUAS-MCS))	This paper	N/A

pF-MCS-HA-StreIII-Bcl2 (StrepIII-tagged Bcl-2 overexpression plasmid, cloned from from pF-5xUAS-MCS))	This paper	N/A

Supplementary Table 7: Software.

NAME	SOURCE	VERSION
FlowJo version 10.8.1	FlowJo LLC (BD)	10.8.1
ImageJ Fiji	[16]	1.53t
XF Wave Seahorse Online Analysis	Agilent	N/A
ZEN Blue and Black Software	Zeiss	2.0
Incucyte® Base Software	Sartorius	2021B
Prism	GraphPad	8
R Studio	RCore Team	4.1.1
R package Seurat version	[17]	4.1.0; RRID:SCR_016341
R package clusterProfiler version	[18,19]	4.0.5; RRID:SCR_016884
R package enrichplot version	[20]	1.12.3
R package DESeq2 version	[21]	1.32.0; RRID:SCR_015687
R package EnhancedVolcano version	[22]	1.10.0; RRID:SCR_018931
R package pheatmap version	[23]	1.0.12; RRID:SCR_016418
Trimmomatic (within usegalaxy.org, 2022)	[24]	RRID:SCR_011848
STAR (within usegalaxy.org, 2022)	[25]	RRID:SCR_004463
featureCounts (within usegalaxy.org, 2022)	[26]	RRID:SCR_012919
FastQC, MultiQC (within usegalaxy.org, 2022)	[27]	RRID:SCR_014583; RRID:SCR_014982

Supplementary Table 8: Chemicals/Drugs.

REAGENT	SOURCE	IDENTIFIER
2-Deoxy-glucose	Sigma-Aldrich	Cat: #D3179; CAS: 154-17-6
4-Hydroxytamoxifen	Sigma-Aldrich	Cat: #H7904; CAS: 68047-06-3
Acridine Orange	Sigma-Aldrich	Cat: #A6014; CAS: 10127-02-3
Actinomycin D	Sigma-Aldrich	Cat: #A1410; CAS: 5076-0
AnnexinV-FITC	Our lab	N/A
Acetaminophen (APAP)	Sigma-Aldrich	Cat: #A7085; CAS: 103-90-2
Bafilomycin A1	Calbiochem	Cat: #196000; CAS: 88899-55-2
Cis-Diamineplatinum(II)dichloride (cisplatin)	Sigma-Aldrich	Cat: #P4394; CAS: 15663-27-1
D-Galactosamine (GalN)	Sigma-Aldrich	Cat: #G0500; CAS: 1772-03-8
D-(+)-Glucose	Sigma-Aldrich	Cat: #16325; CAS: 50-99-7
Glutathione Reductase	Sigma-Aldrich	Cat: #G3664; CAS: 9001-48-3

Hoechst 33342	Invitrogen	Cat: #H3570;<br>CAS: 23491-52-3
JNK Inhibitor V (JNK-V)	Enzo Life Sciences	Cat: # ALX-270-443;<br>CAS: 345987-15-7
LysoTracker™ Green DND-26	Invitrogen	Cat: #L7526;<br>CAS: 67-68-5
Magic Red™ Cathepsin B Kit (Magic Red Substrate (MR-RR$_2$)	BIO-RAD	Cat: #ICT937
MitoSOX™ Red	Invitrogen	Cat: #M36008
MitoTracker™ Green FM	Invitrogen	Cat: #M7514;<br>CAS: 201860-17-5
Murine recombinant TNF-alpha	Peprotech	Cat: # 315-01A;<br>P06804
peqGOLD TriFast™	VWR	Cat: #30-2030
Propidium iodide	Sigma-Aldrich	Cat: #P4864;<br>CAS: 25535-16-4
Rotenone	Sigma-Aldrich	Cat: #R8875;<br>CAS: 83-79-4
Tetramethylrhodamin-ethylester-perchlorat (TMRE)	Sigma-Adrich	Cat: #87917;<br>CAS: 115532-52-0
TRIzol™ Reagent	Invitrogen	Cat: #15596026
Z-VAD-FMK	Enzo Life Sciences	Cat: #ALX-260-020;<br>CAS: 220644-02-0
2-Deoxy-glucose	Sigma-Aldrich	Cat: #D3179;<br>CAS: 154-17-6
4-Hydroxytamoxifen	Sigma-Aldrich	Cat: #H7904;<br>CAS: 68047-06-3
Acridine Orange	Sigma-Aldrich	Cat: #A6014;<br>CAS: 10127-02-3
Actinomycin D	Sigma-Aldrich	Cat: #A1410;<br>CAS: 5076-0
AnnexinV-FITC	Our lab	N/A
Acetaminophen (APAP)	Sigma-Aldrich	Cat: #A7085;<br>CAS: 103-90-2
Bafilomycin A1	Calbiochem	Cat: #196000;<br>CAS: 88899-55-2
Cis-Diamineplatinum(II)dichloride (cisplatin)	Sigma-Aldrich	Cat: #P4394;<br>CAS: 15663-27-1
D-Galactosamine (GalN)	Sigma-Aldrich	Cat: #G0500;<br>CAS: 1772-03-8
D-(+)-Glucose	Sigma-Aldrich	Cat: #16325;<br>CAS: 50-99-7
Glutathione Reductase	Sigma-Aldrich	Cat: #G3664;<br>CAS: 9001-48-3
Hoechst 33342	Invitrogen	Cat: #H3570;<br>CAS: 23491-52-3
JNK Inhibitor V (JNK-V)	Enzo Life Sciences	Cat: # ALX-270-443;<br>CAS: 345987-15-7
LysoTracker™ Green DND-26	Invitrogen	Cat: #L7526;<br>CAS: 67-68-5
Magic Red™ Cathepsin B Kit (Magic Red Substrate (MR-RR$_2$)	BIO-RAD	Cat: #ICT937
MitoSOX™ Red	Invitrogen	Cat: #M36008
MitoTracker™ Green FM	Invitrogen	Cat: #M7514;<br>CAS: 201860-17-5
Murine recombinant TNF-alpha	Peprotech	Cat: # 315-01A;<br>P06804
peqGOLD TriFast™	VWR	Cat: #30-2030
Propidium iodide	Sigma-Aldrich	Cat: #P4864;<br>CAS: 25535-16-4

Rotenone	Sigma-Aldrich	Cat: #R8875;<br>CAS: 83-79-4
Tetramethylrhodamin-ethylester-perchlorat (TMRE)	Sigma-Adrich	Cat: #87917;<br>CAS: 115532-52-0
TRIzol^{TM} Reagent	Invitrogen	Cat: #15596026
Z-VAD-FMK	Enzo Life Sciences	Cat: #ALX-260-020;<br>CAS: 220644-02-0

Supplementary Table 9: Commercial Kits/Assays.

NAME	SOURCE	IDENTIFIER
Seahorse XF Cell Mito Stress Test Kit	Agilent	Cat: #103010-100
PierceTM BCA Protein Assay Kit	Thermo Fisher	Cat: #23225
CellTiter-Glo® 2.0 Cell Viability ATP Assay	Promega	Cat: #9242
ImmPACT DAB Substrate, Peroxidase (HRP)	Vector Laboratories	Cat: #SK-4105
Fast SYBR® Green Master Mix	Applied Biosystems	Cat: #4385614
Seahorse XF Real-Time ATP Rate Assay Kit	Agilent	Cat: #103592-100
LAC 142	Diaglobal	Cat: #LAC 142
GlucoCheck Excellent	Aktivmed	N/A
ALT/GPT Reflotron® Test	Roche Diagnostics	Cat: #10745138202

Supplementary Table 10: Technical Devices.

NAME	SOURCE
Cellomics ArrayScan VTI HCS Reader	Thermo Fisher
Incucyte® SX1	Sartorius
LSM 880 with AiryscanFast	Zeiss
AxioObserver	Zeiss
Reflotron® Plus System	Roche Diagnostics
Seahorse XFe24 Analyzer	Agilent

REFERENCES

1 Kolodziejczyk AA, Federici S, Zmora N, Mohapatra G, Dori-Bachash M, Hornstein S *et al.* Acute liver failure is regulated by MYC- and microbiome-dependent programs. *Nat Med* 2020; **26**: 1899–1911.

2 Mossanen JC, Tacke F. Acetaminophen-induced acute liver injury in mice. *Lab Anim* 2015; **49**: 30–6.

3 Portmann S, Fahrner R, Lechleiter A, Keogh A, Overney S, Laemmle A *et al.* Antitumor effect of SIRT1 inhibition in human HCC tumor models in vitro and in vivo. *Mol Cancer Ther* 2013; **12**: 499–508.

4 Sanjana NE, Shalem O, Zhang F. Improved vectors and genome-wide libraries for CRISPR screening. *Nat Methods* 2014; **11**: 783–784.

5 Vince JE, Wong WWL, Khan N, Feltham R, Chau D, Ahmed AU *et al.* IAP Antagonists Target cIAP1 to Induce TNFα-Dependent Apoptosis. *Cell* 2007; **131**: 682–693.

6 Moreno-Villanueva M, Pfeiffer R, Sindlinger T, Leake A, Müller M, Kirkwood TBL *et al.* A modified and automated version of the 'Fluorimetric Detection of Alkaline DNA Unwinding' method to quantify formation and repair of DNA strand breaks. *BMC Biotechnol* 2009; **9**: 39.

7 Villunger A, Michalak EM, Coultas L, Müllauer F, Böck G, Ausserlechner MJ *et al.* p53- and Drug-Induced Apoptotic Responses Mediated by BH3-Only Proteins Puma and Noxa. *Science (80-)* 2003; **302**: 1036–1038.

8 Bouillet P, Metcalf D, Huang DCS. Proapoptotic Bcl-2 relative Bim required for certain apoptotic responses, leukocyte homeostasis, and to preclude autoimmunity. *Science (80-)* 1999; **286**: 1735–1738.

9 Lowe SW, Schmitt EM, Smith SW, Osborne BA, Jacks T. P53 is required for radiation-induced apoptosis in mouse thymocytes. *Nature* 1993; **362**: 847–849.

10 Brokatzky D, Dörflinger B, Haimovici A, Weber A, Kirschnek S, Vier J *et al.* A non-death function of the mitochondrial apoptosis apparatus in immunity. *EMBO J* 2019; **38**: 1–13.

11 Chandra R, Engeln M, Schiefer C, Patton MH, Martin JA, Werner CT *et al.* Drp1 Mitochondrial Fission in D1 Neurons Mediates Behavioral and Cellular Plasticity during Early Cocaine Abstinence. *Neuron* 2017; **96**: 1327-1341.e6.

12 Zhang Y, Yao Y, Qiu X, Wang G, Hu Z, Chen S *et al.* Listeria hijacks host mitophagy through a novel mitophagy receptor to evade killing. *Nat Immunol* 2019; **20**: 433–446.

13 Li L, Pan R, Li R, Niemann B, Aurich A, Chen Y *et al.* Mitochondrial Biogenesis and PGC-1alpha Deacetylation by Physical Activity: Intact Adipocytokine Signaling Is Required. *Diabetes* 2011; **60**: 157–167.

14 Shalem O, Sanjana NE, Hartenian E, Shi X, Scott DA, Mikkelsen TS *et al.* Genome-Scale CRISPR-Cas9 Knockout Screening in Human Cells. *Science (80-)* 2014; **343**: 84–87.

15 Louvion JF, Havaux-Copf B, Picard D. Fusion of GAL4-VP16 to a steroid-binding domain provides a tool for gratuitous induction of galactose-responsive genes in yeast. *Gene* 1993; **131**: 129–134.

16 Schneider CA., Rasband WS., W. EK. NIH Image to ImageJ: 25 years of Image Analysis. *Nat Methods* 2012; **9**: 671–675.

17 Butler A, Hoffman P, Smibert P, Papalexi E, Satija R. Integrating single-cell transcriptomic data across different conditions, technologies, and species. *Nat Biotechnol*

2018; **36**: 411–420.

18 Wu T, Hu E, Xu S, Chen M, Guo P, Dai Z *et al.* clusterProfiler 4.0: A universal enrichment tool for interpreting omics data. *Innov* 2021; **2**: 100141.

19 Yu G, Wang L-G, Han Y, He Q-Y. clusterProfiler: an R Package for Comparing Biological Themes Among Gene Clusters. *Omi A J Integr Biol* 2012; **16**: 284–287.

20 Yu G. enrichplot: Visualization of Functional Enrichment Result. 2022.https://yulab-smu.top/biomedical-knowledge-mining-book/.

21 Love MI, Huber W, Anders S. Moderated estimation of fold change and dispersion for RNA-seq data with DESeq2. *Genome Biol* 2014; **15**: 550.

22 Blighe K, Rana S, Lewis M. EnhancedVolcano: publication-ready volcano plots with enhanced colouring and labeling. 2021.https://github.com/kevinblighe/EnhancedVolcano.

23 Kolde R. pheatmap: Pretty Heatmaps. 2019.https://cran.r-project.org/web/packages/pheatmap/index.html.

24 Bolger AM, Lohse M, Usadel B. Trimmomatic: a flexible trimmer for Illumina sequence data. *Bioinformatics* 2014; **30**: 2114–2120.

25 Dobin A, Davis CA, Schlesinger F, Drenkow J, Zaleski C, Jha S *et al.* STAR: ultrafast universal RNA-seq aligner. *Bioinformatics* 2013; **29**: 15–21.

26 Liao Y, Smyth GK, Shi W. featureCounts: an efficient general purpose program for assigning sequence reads to genomic features. *Bioinformatics* 2014; **30**: 923–930.

27 Ewels P, Magnusson M, Lundin S, Käller M. MultiQC: summarize analysis results for multiple tools and samples in a single report. *Bioinformatics* 2016; **32**: 3047–3048.
